# Unripe *Rubus coreanus* Miquel Extract Containing Ellagic Acid Promotes Lipolysis and Thermogenesis In Vitro and In Vivo

**DOI:** 10.3390/molecules25245954

**Published:** 2020-12-16

**Authors:** Kyeong Jo Kim, Eui-Seon Jeong, Ki Hoon Lee, Ju-Ryun Na, Soyi Park, Jin Seok Kim, Chang-Su Na, Young Ran Kim, Sunoh Kim

**Affiliations:** 1 Central R&D Center, Bioresources and Technology (B&Tech) Co., Ltd., Gwangju 61239, Korea; kkkjzzang@nate.com (K.J.K.); 22c_goldskin@naver.com (E.-S.J.); leekh7@epost.kr (K.H.L.); ryun1225@daum.net (J.-R.N.); soyipark@hanmail.net (S.P.); keki2000@naver.com (J.S.K.); 2 College of Pharmacy and Research Institute of Drug Development, Chonnam National University, Gwangju 500-757, Korea; 3 College of Korean Medicine, Dongshin University, Naju-si, Jeollanam-do 58245, Korea; nakugi@daum.net

**Keywords:** unripe *Rubus coreanus*, ellagic acid, antiobesity, PPARα, ATGL, UCP1, PGC1α

## Abstract

Previously, we demonstrated that a 5% ethanol extract of unripe *Rubus coreanus* (5-*u*RCK) and ellagic acid has hypocholesterolemic and antiobesity activity, at least partially mediated by the downregulation of adipogenic and lipogenic gene expression in high-fat diet (HFD)-fed animals. The present study investigated the thermogenic and lipolytic antiobesity effects of 5-*u*RCK and ellagic acid in HFD-induced obese C57BL/6 mice and explored its mechanism of action. Mice fed an HFD received 5-*u*RCK or ellagic acid as a post-treatment or pretreatment. Both post-treated and pretreated mice showed significant reductions in body weight and adipose tissue mass compared to the HFD-fed mice. The protein levels of lipolysis-associated proteins, such as adipose triglyceride lipase (ATGL), phosphorylated hormone-sensitive lipase (p-HSL), and perilipin1 (PLIN1), were significantly increased in both the 5-*u*RCK- and ellagic acid-treated mouse epididymal white adipose tissue (eWAT). Additionally, thermogenesis-associated proteins, such as peroxisome proliferator-activated receptor α (PPARα), carnitine palmitoyl transferase-1 (CPT1), uncoupling protein 1 (UCP1), and peroxisome proliferator-activated receptor-γ coactivator-1α (PGC1α), in inguinal white adipose tissue (ingWAT) were clearly increased in both the 5-*u*RCK- and ellagic acid-treated mice compared to HFD-fed mice. These results suggest that 5-*u*RCK and ellagic acid are effective for suppressing body weight gain and enhancing the lipid profile.

## 1. Introduction

Obesity is a major risk factor for cardiovascular diseases (CVDs), including diabetes, hypertension, dyslipidemia, and coronary heart disease. It is characterized by an increase in adipocyte cell size (hypertrophy) and adipocyte number (hyperplasia), leading to an abnormal increase in fat mass and excessive fat accumulation in the mesentery, epididymis, and other organs. Many researchers and companies continue to try to develop therapies for obesity control. The Food and Drug Administration (FDA) has approved a number of central nervous system (CNS)-acting antiobesity agents, such as sibutramine, orlistat, lorcaserin, and phentermine/topiramate, and these agents are used clinically [1]. However, although these drugs help reduce body weight, their usages are limited by a variety of adverse effects, such as pulmonary hypertension, cardiovascular toxicity, stroke, nonfatal cardiovascular events, and neuropsychiatric issues [2]. Therefore, new medicines based on natural products offer a new approach to the development of more effective novel therapeutic agents. Hundreds of studies have suggested that a large range of phytochemicals in fruits have significant potential in the management of obesity in both in vitro and in vivo models [3,4].

The *Rubus* genus comprises thousands of species of blackberries and raspberries grown worldwide, and species in this genus have been investigated as novel therapeutic agents for metabolic syndrome because of their beneficial effects [5]. One such species, *Rubus coreanus* Miquel, which is native to Eastern Asia, has been used as a traditional alternative medicine to manage impotence, spermatorrhea, enuresis, asthma, and allergic diseases [6]. In fact, the small fruits of this species contain many nutrients, such as minerals, vitamins, and sugars, as well as phenolic compounds, including phenolic acids, flavonoids, and tannins [7]. Thus, many beneficial effects of *R. coreanus* Miquel, such as its antitumor, antioxidative, anti-inflammatory, and antiobesity effects, have been reported [8,9]. One of the major active compounds in *R. coreanus* is ellagic acid (EA), which has antiobesity and antioxidant properties [10,11]. The unripe fruits of *R. coreanus* Miquel are usually used as traditional medicine and might be more therapeutic than the ripe fruits. A recent report revealed that the unripe fruits of *R. coreanus* have a higher concentration of ellagic acid than the ripe fruits [12], which might be the reason why unripe fruits are traditionally used.

Peroxisome proliferator-activated receptor γ (PPARγ) is essential for both adipocyte differentiation and the maintenance of mature adipocytes; indeed, PPARγ-deficient embryonic stem cells lose the capability to differentiate into adipocytes [13,14]. Three members of the CCAAT enhancer-binding protein (C/EBP) family, C/EBPα, C/EBPβ, and C/EBPγ, are also critical for the differentiation and metabolism of adipose tissue [15,16,17,18,19]. In addition, sterol regulatory element binding protein 1 (SREBP1) also has a role in adipogenesis and lipogenesis by regulating the expression of lipogenic proteins, including acetyl-CoA carboxylase 1 (ACC1) and fatty acid synthase (FAS) [20]. Our previous study demonstrated the antiobesity [21,22] and antihypercholesterolemic effects [23,24] of a 5% ethanol extract of unripe *R. coreanus* (5-*u*RCK, standardized ethanol extract) cultivated in Korea in high-fat diet (HFD)-fed and high-cholesterol diet (HCD)-fed mice; moreover, we suggested that six bioactive compounds (ellagic acid, erycibelline, 5-hydroxy-2-pyridinemethanol, m-hydroxyphenylglycine, p-aminophenol, and 4-hydroxycoumarin) contribute to the decreased lipid accumulation in murine 3T3-L1 adipocytes demonstrated in another report [22]. Among them, ellagic acid decreased lipid accumulation and expression levels of the key adipogenic genes PPARγ, C/EBPα, SREBP-1c, ACC, and FAS to the greatest extent in the HFD-fed mouse model. Furthermore, 5-*u*RCK and ellagic acid treatment significantly increased the expression of AMP-activated protein kinase (AMPK), p-AMPK, p-HMGCR, and insulin-induced gene 1 (INSIG-1) in a dose-dependent manner and decreased the expression of HMGCR and mSREBP-2 in an HCD-induced hypercholesterolemia rat model [24]. Most recently, according to our clinical trial in humans, 5-*u*RCK supplementation improved the lipid profile, including a reduction in low-density lipoprotein cholesterol (LDL-C), non-high-density lipoprotein cholesterol (non-HDL-C), apolipoprotein (Apo) B, and oxidized LDL levels [25]. Thus, our previous studies in animal models and clinical trials demonstrated that 5-*u*RCK exerts antihypercholesterolemic and antiobesity effects. However, the molecular mechanism of the lipolytic and browning effects of 5-*u*RCK and ellagic acid has not yet been explored.

The major contribution of white adipose tissue (WAT) to whole-body energy metabolism is lipolysis, in which stored triglycerides (TGs) are released as nonesterified free fatty acids (FFAs) and glycerol [26]. Furthermore, brown adipose tissue (BAT) dissipates energy as heat and hence has an important therapeutic capacity for obesity [27]. The development of brown-like adipocytes (also called beige) is another attractive target for obesity treatment. Therefore, this study hypothesized that 5-*u*RCK consumption modulates lipolytic and browning activity in vitro and in HFD-induced obese animal models. The expression of proteins involved in lipolysis, such as adipose triglyceride lipase (ATGL), phosphorylated hormone-sensitive lipase (p-HSL) and perilipin1 (PLIN1), was analyzed in WAT. Additionally, peroxisome proliferator-activated receptor α (PPARα), carnitine palmitoyl transferase-1 (CPT1), uncoupling protein 1 (UCP1) and peroxisome proliferator-activated receptor-γ coactivator-1α (PGC1α) protein levels, which are related to thermogenesis, were assessed.

## 2. Results

### 2.1. 5-uRCK and Ellagic Acid Improve Lipolysis in Mature Adipocytes

**Figure 1:**
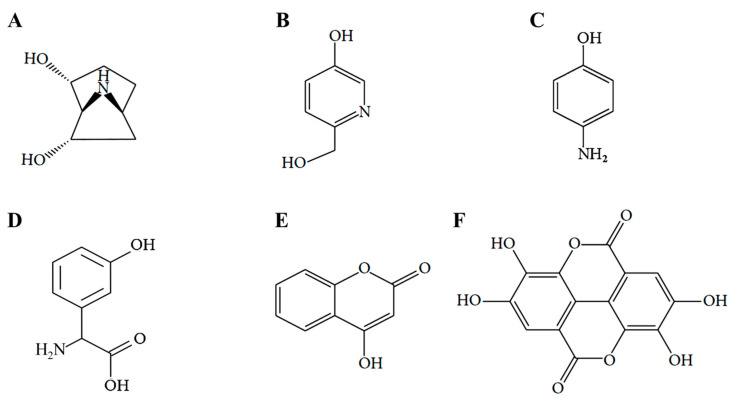
Structural formulas of the six bioactive compounds of 5-*u*RCK. (**A**) erycibelline, (**B**) 5-hydroxy-2-pyridinemethanol, (**C**) p-aminophenol, (**D**) m-hydroxyphenylglycine, (**E**) 4-hydroxycoumarin and (**F**) ellagic acid.

As shown in chemical structure (Figure 1), we previously published the results of 5-*u*RCK compounds (erycibelline, 5-hydroxy-2-pyridinemethanol, p-aminophenol, m-hydroxyphenylglycine, 4-hydroxycoumarin and ellagic acid) analysis [22,24]. We reported the effects of 5-*u*RCK and ellagic acid on the inhibition of lipid accumulation during the differentiation of 3T3-L1 preadipocytes to adipocytes. Furthermore, our previous study reported that 5-*u*RCK and ellagic acid treatment did not affect the viability of 3T3-L1 preadipocytes and mature adipocytes [21,22,23]. In addition, all our previous studies have indicated that 5-*u*RCK has the greatest antiobesity and antihypercholesterolemic effects of all tested *u*RCK extracts prepared by various extraction methods (water, 5% ethanol, 30% ethanol and 70% ethanol) [21,22,23,24,25]. Quantitative analysis by HPLC showed that the aqueous extract and 5-*u*RCK contained 14–16 mg/g and 18–21 mg/g ellagic acid, respectively [24]. Other compounds, such as erycibelline, 5-hydroxy-2-pyridinemethanol, m-hydroxyphenylglycine, p-aminophenol, and 4-hydroxycoumarin were present at very low concentrations in both aqueous extract and 5-*u*RCK. Based on these results, we investigated the effect of 5-*u*RCK and ellagic acid on the activation of lipolysis in differentiated mature adipocytes.

**Figure 2:**
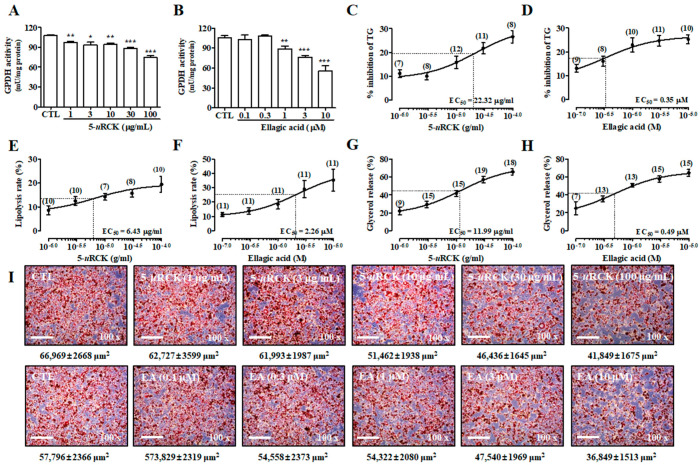
Effects of 5% ethanol extract of unripe *Rubus coreanus* (5-*u*RCK) and ellagic acid in mature 3T3-L1 adipocytes. Differentiated 3T3-L1 adipocytes were incubated with 5-*u*RCK (1–100 μg/mL) or ellagic acid (0.1–10 μM) for 3 days. Inhibitory effects of 5-*u*RCK (**A**) and ellagic acid (**B**) against glycerol 3-phosphate dehydrogenase (GPDH)-specific activity. Effects of 5-*u*RCK (**C**) and ellagic acid (**D**) on triglyceride (TG) accumulation in mature 3T3-L1 adipocytes. Quantification of the lipolysis rate in mature adipocytes with 5-uRCK (**E**) and ellagic acid (**F**). Mature 3T3-L1 cells were incubated with various concentrations of 5-*u*RCK (**G**) or ellagic acid (**H**) for 24 h. Values are expressed as the mean amount (±SE) of glycerol released per well as a percentage of the control value. Numbers above each point indicate the n-number at each concentration. (**I**) Matured 3T3-L1 adipocytes were stained with Oil Red O. The size of Oil Red O-stained lipid droplets (μm^2^) was calculated using ImageJ software version 1.48 s (National Institutes of Health, Bethesda, MD, USA). The results are presented as the mean ± SE of at least three independent experiments, each performed in triplicate (n = 3). * *p* < 0.05, ** *p* < 0.01 and *** *p* < 0.001 versus control.

First, we measured the effects of 5-*u*RCK and ellagic acid on glycerol 3-phosphate dehydrogenase (GPDH) activity and TG levels in mature adipocytes. GPDH plays an important role in the conversion of glycerol to TG, especially in adipocytes, and it is a marker of the late phase of adipocyte differentiation [28]. The effects of 5-*u*RCK and ellagic acid on GPDH activity in mature 3T3-L1 adipocytes are shown in Figure 2A,B. Both 5-*u*RCK (1–100 μg/mL) and ellagic acid (0.1–10 μM) significantly inhibited GPDH activity in a dose-dependent manner after incubation for 3 days. Furthermore, high doses of 100 μg/mL 5-*u*RCK and 10 μM ellagic acid inhibited TG content by 26.59 ± 2.61% and 25.27 ± 1.82%, respectively after incubation for 3 days, compared to the untreated control (Figure 2C,D). We assessed the lipolysis of mature adipocytes by microscopy using Oil Red O (ORO) staining and measured the lipid content. As shown in Figure 2E,I, 5-*u*RCK reduced the number of lipid droplets as marked by positive ORO staining during microscopy and increased the quantitative measurement of lipolysis. In accordance with these results, 5-*u*RCK significantly reduced lipid contents by 8.6%, 12.5%, 14.3%, 15.8%, and 19.5% at concentrations of 1, 3, 10, 30, and 100 μg/mL, respectively. Additionally, the accumulation of ORO-stained lipid droplets was visible in most of the untreated control 3T3-L1 adipocytes; however, the number of detectable stained droplets decreased in the presence of ellagic acid in mature adipocytes (Figure 2F). To investigate whether 5-*u*RCK or ellagic acid regulates triacylglycerol metabolism in adipocytes, differentiated 3T3-L1 cells were evaluated for lipolysis after a 24 h treatment. As depicted in Figure 2G,H, incubation with 5-*u*RCK or ellagic acid increased the basal level of glycerol released into the media in a dose-dependent manner after 24 h of treatment.

### 2.2. 5-uRCK Reduces Body Weight Gain in 16-Week Long-Term HFD-Fed Mice

**Figure 3:**
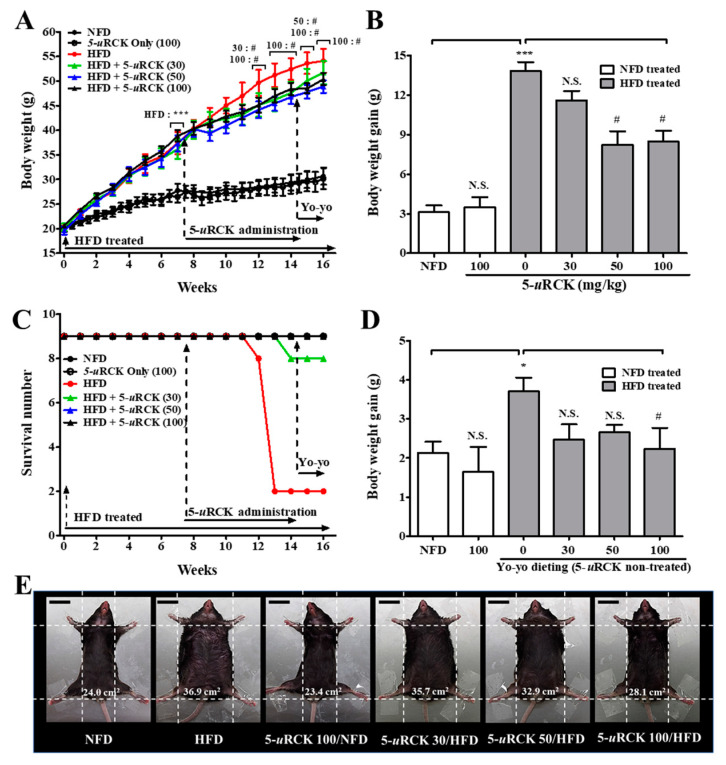
Changes in body weight throughout the experimental period. (**A**) Body weight was monitored three times per week for 16 weeks. (**B**) Body weight gain was measured from the 8th week until the 14th week. (**C**) Effect of 5-*u*RCK treatment after obesity development on the survival rate. (**D**) Body weight gain was measured from the 14th week until the 16th week. (**E**) Phenotype of representative mice from each group. The body width of each group of mice was checked before sacrifice. The scale bars indicate 20 mm. The results are presented as the mean ± SD (n = 9). * *p* < 0.05 and *** *p* < 0.001 vs. normal-fat diet (NFD) group; ^#^ *p* < 0.05 vs. high-fat diet (HFD) group. N.S., not significant.

We next investigated in vivo the antiobesity action of 5-*u*RCK in an HFD-induced obese mouse model (Figure 3). Initial body weights at the beginning of the experiment were not different among the six groups. HFD-induced obese mice were obtained after 7 weeks of treatment when the weight difference was over 25% (*p* < 0.001). After 16 weeks of treatment, the mice fed an HFD developed 43% higher body weights than the normal-fat diet (NFD) control mice (30.69 g compared with 54.11 g). Body weight loss was not significant in 5-*u*RCK (100 mg/kg/day)-treated animals fed the NFD diet compared with the NFD-fed animals (Figure 3A,B). These results suggest that treatment with 5-*u*RCK did not induce in vivo toxicity in mice. Interestingly, both the animals fed an HFD + 5-*u*RCK (50 mg/kg/day) and an HFD + 5-*u*RCK (100 mg/kg/day) for 7 weeks showed a reduction in body weight of approximately 40% compared with animals in the HFD group (*p* < 0.05). The reduction in body weight was unlikely to be due to changes in food intake because 5-*u*RCK treatment did not significantly affect average food intake in either NFD- or HFD-fed mice (data not shown). Food intake results were also similar to our previously reported results [21,22,23,24]. As shown Figure 2C, the mortality rates of the NFD-fed group and 5-*u*RCK-treated group fed the NFD diet were 0% at 16 weeks. However, the mortality rate of the HFD-fed group was 22% at 14 weeks. The survival rates of the 5-*u*RCK 30, 5-*u*RCK 50, and 5-*u*RCK 100 groups fed the HFD diet were 89%, 100%, and 100% at 14 weeks, respectively. In addition, yo-yo dieting significantly reduced body weight gain in the 5-*u*RCK (100 mg/kg)-treated mice compared to untreated HFD mice (*p* < 0.05) (Figure 3D). Representative images of the mice are shown in Figure 3E. Mice fed an HFD were rounder and larger in size than those in the NFD group; moreover, 5-*u*RCK (50 and 100 mg/kg)-treated mice had a slimmer appearance than the HFD-fed mice, which might be associated with reduced fat accumulation.

### 2.3. Post-Treatment with 5-uRCK Reduced Body Weight Gain and Fat Accumulation in HFD-Fed Mice at 8 Weeks

**Figure 4:**
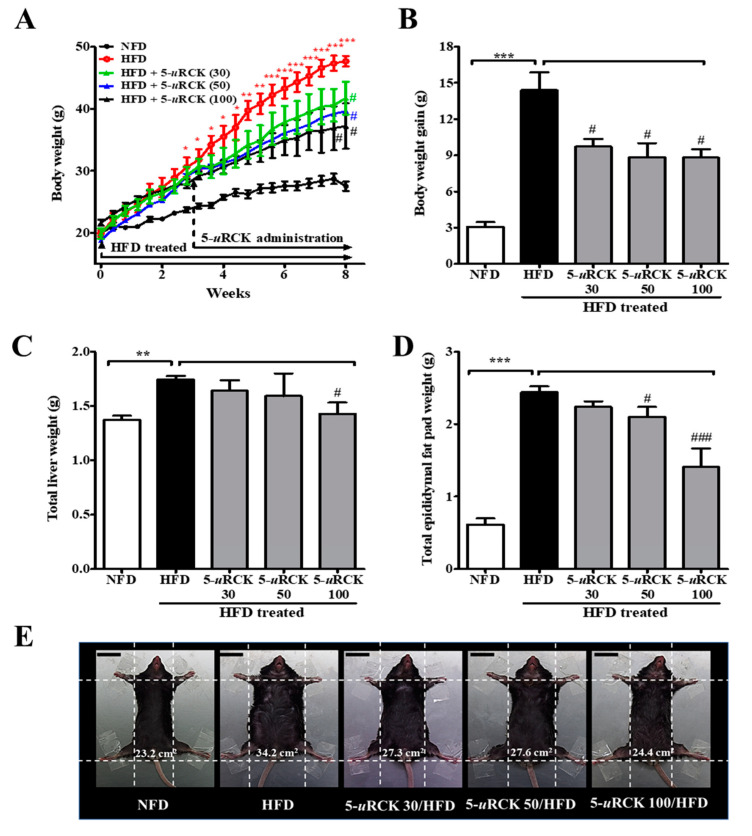
Effects of 5-*u*RCK on body weight changes, liver weight, and epididymal adipose tissue weight in HFD-induced obese mice. (**A**) Body weight was monitored three times per week for 8 weeks. (**B**) Body weight gain was measured from the 3rd week until the 8th week. (**C**) The weight of total liver tissue. (**D**) The weight of the epididymal fat pad; (**E**) Phenotype of representative mice from each group. The body width of each group of mice was checked before sacrifice. The scale bars indicate 20 mm. The results are presented as the mean ± SD (n = 8). * *p* < 0.05, ** *p* < 0.01 and *** *p* < 0.001 vs. NFD group; ^#^ *p* < 0.05 and ^###^ *p* < 0.001 vs. HFD group.

The effects of dietary 5-*u*RCK on body weight change are shown in Figure 4. Initial body weights at the beginning of the experiment were not different among the five groups. Mice were maintained on an HFD for 3 weeks and then treated with 5-*u*RCK plus the HFD for another 5 weeks. In the three successive weeks of HFD feeding, the body weight gain in the HFD group was significantly higher (*p* < 0.05) than that in the NFD group. Furthermore, mice in the HFD group showed a significant increase (*p* < 0.001) in body weight compared with those in the NFD control group after 8 weeks of treatment (Figure 4A,B). However, the administration of 5-*u*RCK at 30, 50, or 100 mg/kg daily for 5 weeks resulted in a significant decrease in body weight (*p* < 0.05) compared with mice in the HFD group. The epididymal fat pad and liver weights in the HFD-fed group were higher than those in the NFD-fed control group. The 5-*u*RCK (100 mg/kg)-treated group had significantly (*p* < 0.05) reduced liver weights compared to the HFD group (Figure 4C and Figure S1a). As shown Figure 4D and Figure S1b, the average epididymal fat pad weight of the 5-*u*RCK 50 group (2.10 g, *p* < 0.05) and 5-*u*RCK 100 group (1.41 g, *p* < 0.001) was significantly lower than that of the HFD group (2.44 g). No abnormal clinical signs were observed during the experimental periods. Additionally, there were no significant differences among the HFD feeding groups in food intake (data not shown). Food intake results were also similar to those previously reported [21,22,23,24]. Figure 4E shows the morphology of the mice in different groups before sacrifice. Mice fed an HFD were rounder and larger in size than those fed an NFD. Body width was reduced in the mice treated with 5-*u*RCK, which might be associated with reduced fat accumulation.

### 2.4. Post-Treatment with 5-uRCK Ameliorated the Lipid Profile in Mice after 8 Weeks of HFD Feeding

At the end of the treatment, effects on plasma lipid parameters—TG, total cholesterol (TC), HDL-C, and LDL-C levels—were analyzed and are presented in Table 1. HFD-fed mice had significantly increased levels of several metabolic risk factors, including TG, total TC, and LDL-C. 5-*u*RCK-supplemented HFD mice showed a significant decrease in plasma TG by 10.4% (*p* < 0.05) at a 100 mg/kg/day dose compared with HFD-fed mice. Similarly, TC levels were significantly decreased in the 5-*u*RCK-treated HFD group by 10.7% (*p* < 0.05) at a 100 mg/kg/day dose. However, the TG and TC levels were not significantly different between the HFD group and HFD + 5-*u*RCK 30 or HFD + 5-*u*RCK 50 groups (*p* > 0.05). Next, we determined HDL-C and LDL-C levels in plasma. 5-*u*RCK treatment significantly increased HDL-C levels by 83.3% (*p* < 0.001), 103.6% (*p* < 0.001), and 92.9% (*p* < 0.001) at 30, 50, and 100 mg/kg/day doses, respectively. LDL-C levels were robustly increased in HFD-fed animals (*p* < 0.001), and 5-*u*RCK reduced LDL-C levels by 12.9% (*p* > 0.05), 13.7% (*p* < 0.05), and 21.4% (*p* < 0.01) at 30, 50, and 100 mg/kg/day doses, respectively, compared with those in the HFD diet group.

### 2.5. Post-Treatment with 5-uRCK Lowers Biochemical Parameters of Serum at 8 Weeks of HFD Feeding

As shown in Table 1, aspartate aminotransferase (AST), alanine aminotransferase (ALT), creatinine (CRE), amylase, uric acid (UA), and lactate dehydrogenase (LDH) were then assayed, and the HFD + 5-*u*RCK 100 treatment was found to significantly decrease these levels in the mice (*p* < 0.01, *p* < 0.001, *p* < 0.001, *p* < 0.001, *p* < 0.001 and *p* < 0.001, respectively). To test whether 5-*u*RCK could lower blood glucose levels in HFD-induced obese mice, blood glucose levels were also measured. The HFD-fed mice exhibited higher blood glucose levels than the NFD control mice, and the HFD + 5-*u*RCK-treated mice showed lower glucose levels than the HFD-fed mice, although the difference was not significant (*p* > 0.05).

### 2.6. Effects of Ellagic Acid on Reducing Body Weight Gain in HFD-Fed Mice at 8 Weeks

**Figure 5:**
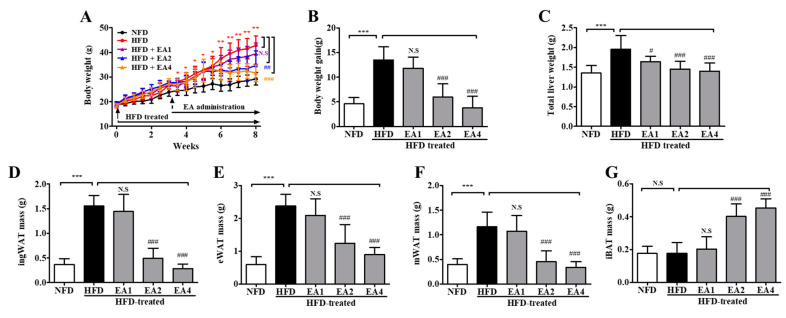
Ellagic acid ameliorates obesity in HFD-fed mice. (**A**) Time course of body weights during the 8-week feeding period. (**B**) Gained body weight, (**C**) total liver weight, (**D**) inguinal white adipose tissue weight, (**E**) epididymal white adipose tissue weight, (**F**) mesenteric white adipose tissue weight, and (**G**) interscapular brown adipose tissue weight. The results are presented as the mean ± SD (n = 8). * *p* < 0.05, ** *p* < 0.01 and *** *p* < 0.001 vs. NFD group; ^#^ *p* < 0.05, ^##^ *p* < 0.01 and ^###^ *p* < 0.001 vs. HFD group. N.S., not significant.

As shown in Figure 5A, consuming an HFD for 3 weeks made mice obese with higher body weights than NFD-fed mice (28.08 ± 0.48 g vs. 23.87 ± 0.54 g, *p* < 0.05). After the induction of obesity in mice fed an HFD, ellagic acid was orally administered once per day for an additional 5 weeks. The administration of ellagic acid at 1, 2, and 4 mg/kg daily for 5 weeks resulted in a significant decrease in body weight (*p* > 0.05, *p* < 0.001 and *p* < 0.001, respectively) compared with mice in the HFD group (Figure 5B). Interestingly, the administration of ellagic acid (4 mg/kg) significantly reduced the body weight of HFD-fed mice from 33.17 ± 1.3 g to 31.53 ± 1.1 g (week 5 versus week 8 of treatment with ellagic acid, *p* < 0.001), which is almost similar to the weights of NFD-fed mice (29.27 ± 0.9 g, 8 weeks). The ellagic acid (1, 2, and 4 mg/kg)-treated group exhibited significantly (*p* < 0.05, *p* < 0.001 and *p* < 0.001, respectively) reduced total liver weight compared to the HFD group (Figure 5C and Figure S2a). The weights of inguinal white adipose tissue (ingWAT), epididymal white adipose tissue (eWAT), and mesenteric white adipose tissue (mWAT) were increased by the HFD (*p* < 0.001 for all), whereas the administration of ellagic acid to HFD-fed mice reduced ingWAT, eWAT, and mWAT weights to a similar weight as each WAT in NFD-fed mice (Figure 5D–F and Figure S2b–d). Interestingly, the relative weight of interscapular brown adipose tissue (iBAT) was increased by 1.2-fold (*p* < 0.001) and 1.5-fold (*p* < 0.001) in the ellagic acid (2 and 4 mg/kg, respectively) supplemented groups compared to that in the HFD group, whereas that in the 1 mg/kg ellagic acid-supplemented group was not different (*p* > 0.05) (Figure 5G and Figure S2e).

### 2.7. Effects of Ellagic Acid on Serum Lipid Levels and Biochemical Parameters in Mice Fed an HFD

To study whether post-treatment with ellagic acid could alleviate metabolic disorders in obese mice, the mice were fed an HFD + ellagic acid diet for 8 weeks. Serum lipid profiles, including TG, TC, LDL-C, and HDL-C, of the five groups of mice are shown in Table 2. HFD feeding increased the serum levels of TG (2.30-fold), TC (1.55-fold), and LDL-C (1.43-fold) compared to NFD feeding. Ellagic acid administration markedly prevented the elevation of serum TG by 52.03% (*p* < 0.001), 28.53% (*p* < 0.001), and 52.07% (*p* < 0.001) (1, 2, and 4 mg/kg, respectively). TC levels were also reduced by ellagic acid treatment by 7.66% (*p* > 0.05), 27.77% (*p* < 0.001), and 27.94% (*p* < 0.001) (1, 2, and 4 mg/kg, respectively). Furthermore, the increased LDL-C level in the HFD group was reduced by treatment with ellagic acid by 10.92% (*p* > 0.05), 31.98% (*p* < 0.01), and 34.36% (*p* < 0.001) (1, 2, and 4 mg/kg, respectively). Additionally, we measured serum ALT and AST, which are clinical markers of liver damage, and our results showed that ellagic acid significantly reduced the HFD-induced increases in AST and ALT serum levels compared with those in HFD-fed mice. The ellagic acid-treated groups also had a significant reduction in CRE, amylase, and LDH levels compared with the HFD group. Moreover, the uric acid (UA) level was higher in the HFD group than in the NFD group, but these levels were not significantly different between the ellagic acid-treated group and the HFD-fed group. Notably, the administration of ellagic acid at either 1 mg/kg or 4 mg/kg significantly attenuated blood glucose levels compared with those of the HFD group.

### 2.8. Effects of 5-uRCK and Ellagic Acid on Histological Changes in Adipose Tissue and Liver

**Figure 6:**
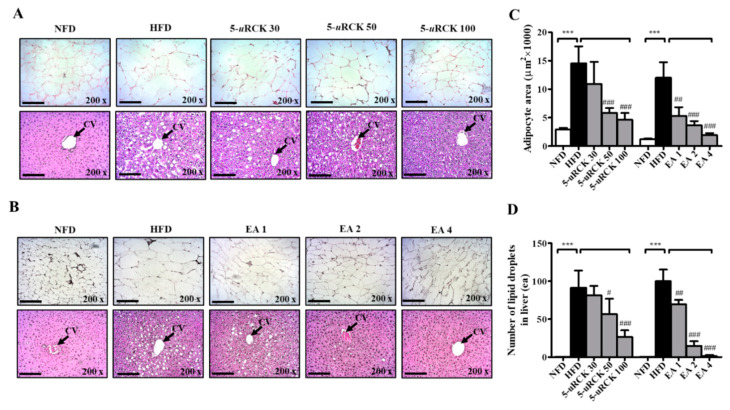
Effects of 5-*u*RCK and ellagic acid on histological changes and lipid accumulation in eWAT and liver tissues of HFD-induced obese mice. Histological changes were determined via hematoxylin and eosin (H&E) staining in epididymal white adipose tissue (eWAT) and liver tissue sections (magnification, ×200) of mice from the (**A**) 5-*u*RCK and (**B**) ellagic acid post-treated mice. The arrow indicates the central vein (CV). (**C**) The adipocyte size was quantified microscopically from representative sections (n = 18). (**D**) The number of lipid deposits in H&E-stained livers was calculated in at least five images in each group. *** *p* < 0.001 vs. NFD group; ^#^ *p* < 0.05, ^##^ *p* < 0.01 and ^###^ *p* < 0.001 vs. HFD group. Scale bars = 200 μm.

To confirm the changes in adipocyte sizes and the number of lipid droplets in the liver, hematoxylin and eosin (H&E) staining of eWAT and liver tissue was performed, and the sizes of adipocytes and lipid droplets in the liver were analyzed. In eWAT, marked fat accumulation was observed in the HFD group (Figure 6A,B), adipocyte sizes were markedly increased (Figure 6C), and these features were inhibited in the 5-*u*RCK- and ellagic acid-treated groups. In all treatment groups, the relative adipocyte size was significantly decreased by treatment with 5-*u*RCK (50 and 100 mg/kg) and ellagic acid (1, 2 and 4 mg/kg). In the HFD group, animals exhibited hepatic lipid accumulation, and 5-*u*RCK and ellagic acid administration also markedly alleviated these conditions (Figure 6D).

### 2.9. Protein Expression Levels for Lipolysis-Related Proteins in Visceral eWAT

**Figure 7:**
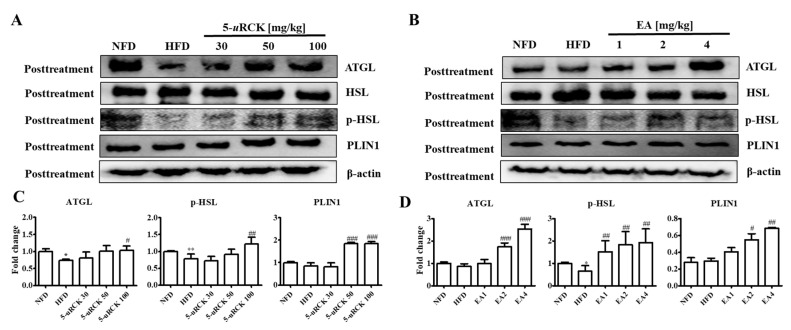
Effect of 5-*u*RCK and ellagic acid supplementation on lipolysis-associated protein in HFD-fed mouse visceral epididymal white adipose tissue (eWAT). Effects of 5-*u*RCK (**A**) and ellagic acid (**B**) on adipose triglyceride lipase (ATGL), hormone-sensitive lipase (HSL), phosphorylated hormone-sensitive lipase (p-HSL), and perilipin1 (PLIN1) expression in eWAT and assessed by western blot analysis. Each value (**C**,**D**) was normalized to β-actin and expressed as the mean ± SD. The levels of p-HSL (Ser563) proteins were normalized to HSL and expressed as the mean ± SD. *P* values for comparisons with control groups (NFD groups) are denoted as * *p* < 0.05 and ** *p* < 0.01, and those for comparisons with HFD groups are denoted as ^#^ *p* < 0.05, ^##^ *p* < 0.01, and ^###^ *p* < 0.001.

Previous results showed that 5-*u*RCK and ellagic acid could decrease and inhibit body weight and adipocyte size compared with the HFD alone. Therefore, we further investigated the molecular mechanism of the lipolytic and thermogenic antiobesity effects of 5-*u*RCK and ellagic acid in adipocytes by protein expression analysis. The protein levels of lipolysis-associated proteins, such as ATGL, p-HSL, and PLIN1, were significantly increased in the post-treatment 5-*u*RCK- or ellagic acid-supplemented groups compared with those in the HFD groups (Figure 7).

### 2.10. Protein Expression Levels for Browning-Related Proteins in Subcutaneous ingWAT

**Figure 8:**
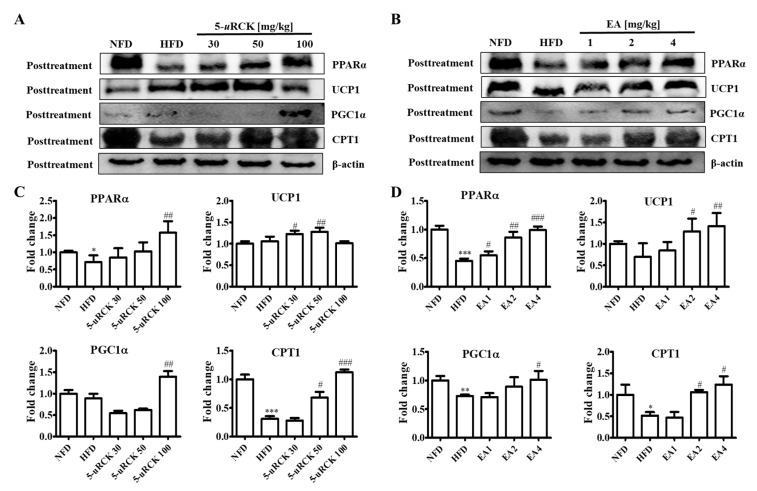
Effect of 5-*u*RCK and ellagic acid supplementation on fat browning-associated protein expression in HFD-fed mice inguinal white adipose tissue (ingWAT). Effects of 5-uRCK (**A**) and ellagic acid (**B**) on peroxisome proliferator-activated receptor α (PPARα), uncoupling protein 1 (UCP1), peroxisome proliferator-activated receptor-γ coactivator-1α (PGC1α), and carnitine palmitoyl transferase-1 (CPT1) expression in the ingWAT and assessed by Western blot analysis. Each value (**C**,**D**) was normalized to β-actin and expressed as the mean ± SD. *P* values for comparisons with control groups (NFD groups) are denoted as * *p* < 0.05, ** *p* < 0.01 and *** *p* < 0.001, and those for comparisons with HFD groups are denoted as ^#^ *p* < 0.05, ^##^ *p* < 0.01, and ^###^ *p* < 0.001.

PPARα, CPT1, UCP1, and PGC1α expression levels were measured to examine the effect of 5-*u*RCK and ellagic acid on thermogenesis. As shown in Figure 8, inguinal white adipose tissue expression of thermogenic proteins such as UCP1 and PGC1α was significantly reduced in the mice fed the HFD compared to those fed the NFD. UCP1 and PGC1α protein levels were significantly increased in the 5-*u*RCK- or ellagic acid-supplemented groups compared with those in the HFD groups. The expression of CPT1, an enzyme involved in mitochondrial fatty acid oxidation, was significantly increased in both the 5-*u*RCK and ellagic acid groups compared with the HFD group. Additionally, the protein expression of PPARα, an upstream transcription factor of CPT1, was markedly decreased in the HFD group but was increased in both the 5-*u*RCK and ellagic acid-post-treated groups.

### 2.11. Pretreatment with 5-uRCK Inhibits HFD-Induced Body Weight Gain

**Figure 9:**
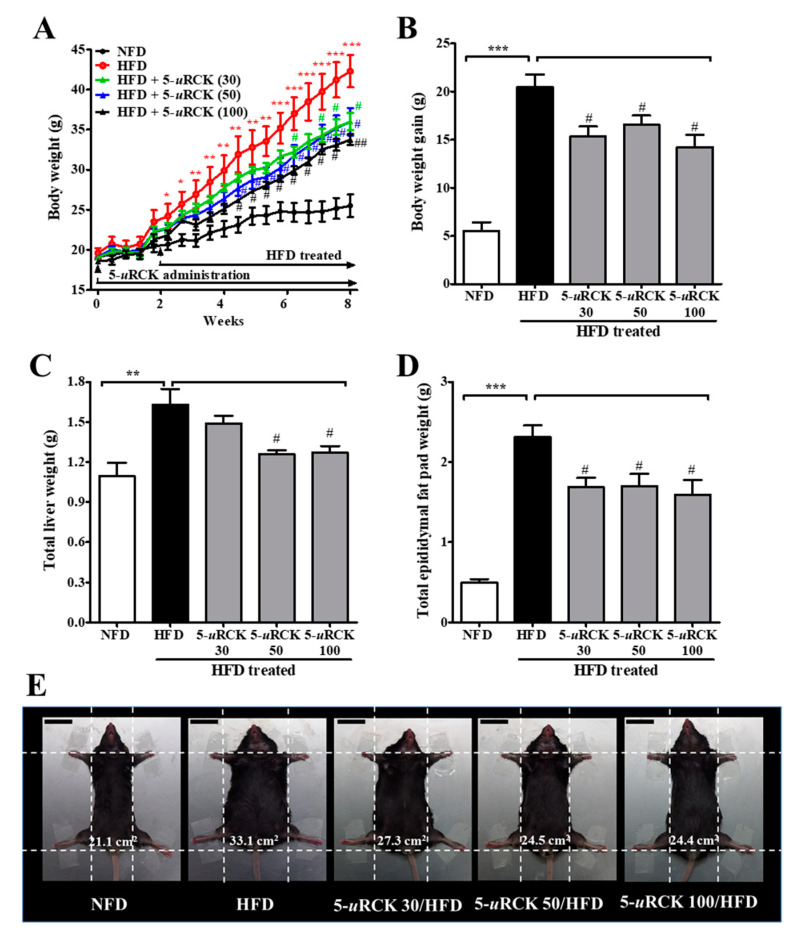
Effect of 5-*u*RCK on body, liver, and epididymal tissue weight in HFD-induced obese mice. (**A**) Body weight was monitored three times per week for 8 weeks. (**B**) Body weight gain was measured from week 0 until the 8th week. (**C**) The weight of total liver tissue; (**D**) The weight of the epididymal fat pad; (**E**) Phenotype of representative mice from each group. The body width of each group of mice was checked before sacrifice. The scale bars indicate 20 mm. The results are presented as the mean ± SD (n = 8). * *p* < 0.05, ** *p* < 0.01 and *** *p* < 0.001 vs. NFD group; ^#^ *p* < 0.05 and ^##^ *p* < 0.01 vs. HFD group.

The effects of dietary 5-*u*RCK on body weight change are shown in Figure 9. In this experiment, mice with similar initial body weights were randomly assigned into five groups (n = 8 per group). By the end of 8 weeks, the final body weight of the HFD group (42.29 ±4.03 g, *p* < 0.001) was significantly higher than that of the NFD group (25.50 ± 3.75 g) (Figure 9A). Pretreatment with 5-*u*RCK (30, 50, and 100 mg/kg) markedly inhibited weight gain in HFD-fed mice (by approximately 24.9%, 18.9%, and 30.1%, respectively) (Figure 9B). Liver weight in mice fed an HFD significantly increased by 48.7% (*p* < 0.01) compared to that in NFD-fed mice, and 5-*u*RCK (30, 50 and 100 mg/kg) inhibited liver weight by 8.70% (*p* > 0.05), 22.82% (*p* < 0.05), and 22.09% (*p* < 0.05), respectively (Figure 9C and Figure S3a). Epididymal adipose tissue weights from mice fed an HFD plus 5-*u*RCK (30, 50, and 100 mg/kg) were significantly inhibited by approximately 27.11%, 26.37%, and 31.09% (*p* < 0.05 for all), respectively (Figure 9D and Figure S3b). Figure 9E shows the morphology of the mice in different groups before sacrifice. Mice fed an HFD were rounder and larger in size than those fed an NFD. Body width was also reduced in the mice treated with 5-*u*RCK, which might be associated with inhibited fat accumulation.

### 2.12. Pretreatment with Ellagic Acid Inhibits Body Weight Gain in HFD-Fed Mice

**Figure 10:**
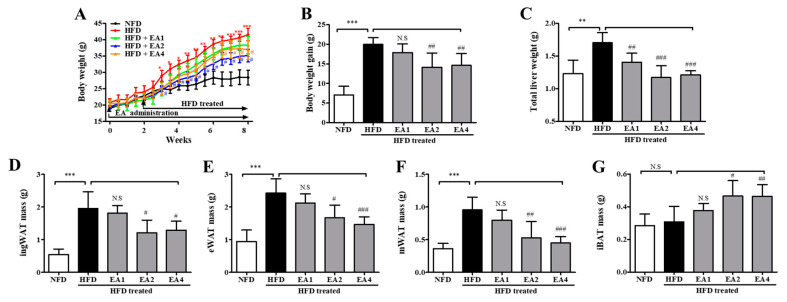
The body and adipose tissue weight changes in mice fed an NFD or an HFD and mice fed an HFD and pretreated with ellagic acid at a dosage of 1, 2, or 4 mg/kg/day. (**A**) Body weights were measured three times per week for 8 weeks. (**B**) Gained body weight, (**C**) total liver weight, (**D**) inguinal white adipose tissue weight, (**E**) epididymal white adipose tissue weight, (**F**) mesenteric white adipose tissue weight, and (**G**) interscapular brown adipose tissue weight. The results are presented as the mean ± SD (n = 8). * *p* < 0.05, ** *p* < 0.01 and *** *p* < 0.001 vs. NFD group; ^#^ *p* < 0.05, ^##^ *p* < 0.01 and ^###^ *p* < 0.001 vs. HFD group. N.S, not significant.

As shown in Figure 10, to investigate the obesity prevention effect of ellagic acid, mice were fed an HFD with ellagic acid (1, 2, and 4 mg/kg, oral gavage daily) for 2 weeks as a pretreatment and then for 6 weeks as a cotreatment. At the end of the 8-week treatment period, HFD mice had a mean body weight gain of 41.57 ± 1.92 g, whereas NFD mice exhibited a gain of 28.42 ± 5.53 g. The mean body weight gain in HFD mice was significantly inhibited by ellagic acid (2 and 4 mg/kg) supplementation (*p* < 0.01 and *p* < 0.01, respectively) (Figure 10A,B). The effects of ellagic acid supplementation on fat accumulation in the liver were investigated. Furthermore, to investigate whether the ellagic acid-induced body weight reduction was associated with the decrease in various fat pads, the pads were dissected and weighed. The results demonstrated that these organs were significantly heavier in HFD mice than in NFD mice and that ellagic acid treatment significantly inhibited fat deposition in these organs compared with those in HFD-fed mice (Figure 10C–F and Figure S4a–d). All WAT weight increases were significantly inhibited by ellagic acid. Interestingly, the relative weight of iBAT was increased by 51.6% (*p* < 0.05) and 50.7% (*p* < 0.01) in the ellagic acid (2 and 4 mg/kg, respectively)-supplemented groups compared to that in the HFD group, whereas the 1 mg/kg ellagic acid-supplemented group was not different (*p* > 0.05) (Figure 10G and Figure S4e).

### 2.13. 5-uRCK and Ellagic Acid Inhibited Serum Lipid Profile Increases in HFD-Induced Obese Mice

HFD-fed mice exhibited significantly higher serum levels of TG (55.15%, *p* < 0.001), TC (87.67%, *p* < 0.001), and LDL-C (79.99%, *p* < 0.001) and significantly lower levels of HDL-C (47.66%, *p* < 0.001) than mice in the NFD group. 5-*u*RCK significantly inhibited TG (30, 50, and 100 mg/kg), TC (50 and 100 mg/kg), and LDL-C (50 and 100 mg/kg) increases and increased HDL-C levels (30, 50, and 100 mg/kg) compared with HFD alone (Table 3). Treatment with 5-*u*RCK significantly and dose-dependently inhibited the increases in the levels of TG, TC, and LDL-C, and at the highest dose examined (100 mg/kg), these lipid profile increases were inhibited by 31.1%, 20.4%, and 23.1%, respectively, compared with those in HFD-fed mice. Moreover, 5-*u*RCK (100 mg/kg) increased HDL-C by 98.2% compared with that in HFD-fed mice. Furthermore, ellagic acid treatment also dose-dependently inhibited serum TG, TC, and LDL-C levels and increased HDL-C levels compared with those in HFD-fed mice (Table 4). Ellagic acid at a dose of 4 mg/kg decreased the mean TG, TC, and LDL-C levels by 31.8% (*p* < 0.001), 19.0% (*p* < 0.001) and 34.7% (*p* < 0.001), respectively, compared with an HFD alone. Moreover, ellagic acid (4 mg/kg) increased HDL-C by 71.9% (*p* < 0.001) compared with an HFD alone.

### 2.14. Pretreatment with 5-uRCK or Ellagic Acid Improves Serum Biochemical Parameters in HFD-Fed Mice

As shown in Table 3, AST, ALT, CRE, amylase, UA, and LDH were assayed, and the HFD + 5-*u*RCK 100 treatment significantly inhibited these in the mice (*p* < 0.001, *p* < 0.001, *p* < 0.001, *p* < 0.05, *p* < 0.001 and *p* < 0.001, respectively). To test whether 5-*u*RCK could lower blood glucose levels in HFD-induced obese mice, blood glucose levels were also measured. The HFD-fed mice exhibited higher blood glucose levels than the NFD control mice, while the HFD + 5-*u*RCK-treated groups showed lower glucose levels than the HFD-fed mice, with no significant difference (*p* > 0.05). In addition, as shown in Table 4, ellagic acid significantly reduced the levels of AST, ALT, CRE, amylase, UA, and LDH in a similar manner to 5-*u*RCK. Interestingly, blood glucose levels were significantly reduced in all groups treated with ellagic acid at 1, 2 and 4 mg/kg (*p* < 0.05, *p* < 0.05 and *p* < 0.001, respectively).

### 2.15. 5-uRCK and Ellagic Acid Suppress Histological Changes in the eWAT and Liver Tissues of HFD-Fed Mice

**Figure 11:**
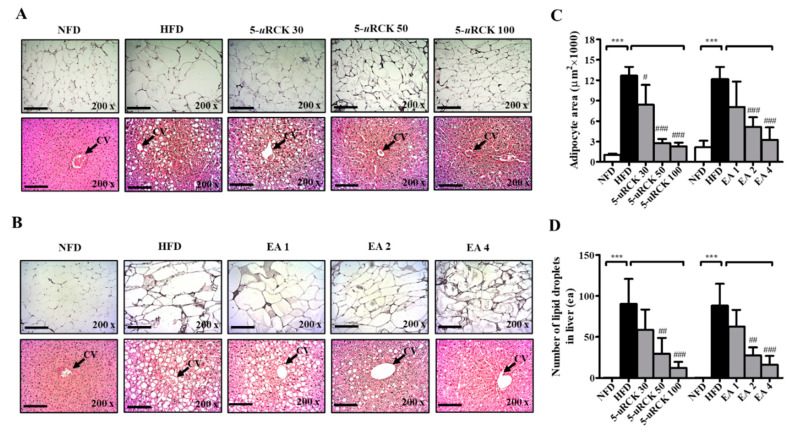
Effects of 5-*u*RCK and ellagic acid on histological changes and lipid accumulation in eWAT and liver tissues of HFD-induced obese mice. Histological changes were determined via H&E staining in eWAT and liver tissue sections (magnification, ×200) of mice from the (**A**) 5-*u*RCK and (**B**) ellagic acid pretreated groups. The arrow indicates the central vein (CV). (**C**) The adipocyte size was quantified microscopically from representative sections (n = 18). (**D**) The number of lipid deposits in H&E-stained livers was calculated in at least five images in each group. *** *p* < 0.001 vs. NFD group; ^#^ *p* < 0.05, ^##^ *p* < 0.01 and ^###^ *p* < 0.001 vs. HFD group. Scale bars = 200 μm.

As shown in Figure 11, H&E staining revealed enlarged or hypertrophic eWAT in the HFD group and smaller eWAT sizes in 5-*u*RCK- or ellagic acid-supplemented mice (Figure 11C). The adipocyte area of the HFD group was approximately 12,000 μm^2^, which was significantly greater than that of the NFD group. Intake of 30–100 mg/kg 5-*u*RCK with an HFD significantly inhibited adipocyte area to approximately 8400, 2800, and 2300 μm^2^. The 2 mg/kg ellagic acid inhibited the area to 5100 μm^2^, and 4 mg/kg ellagic acid significantly inhibited it to 3300 μm^2^, suggesting a dose-dependent effect of 5-*u*RCK and ellagic acid on adipocyte size. In addition, the histological study revealed enlarged hepatocytes, as evidenced by excessive vacuolation in the HFD group compared with the NFD group. As presented in Figure 11D, 5-*u*RCK or ellagic acid markedly reduced the lipid droplet number in the liver tissues of HFD mice.

### 2.16. Protein Expression Levels for Lipolysis-Related Proteins in Visceral eWAT

**Figure 12:**
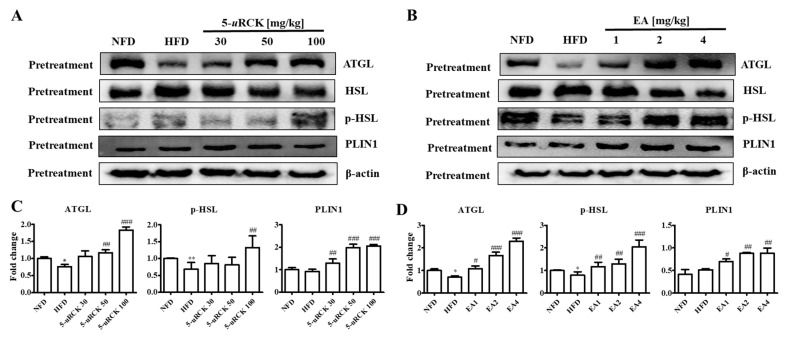
Effect of 5-*u*RCK and ellagic acid supplementation on lipolysis-associated protein in HFD-fed mouse visceral epididymal white adipose tissue (eWAT). Effects of 5-*u*RCK (**A**) and ellagic acid (**B**) on ATGL, HSL, p-HSL, and PLIN1 expression in eWAT and assessed by Western blot analysis. Each value (**C**,**D**) was normalized to β-actin and expressed as the mean ± SD. The levels of p-HSL (Ser563) proteins were normalized to HSL and expressed as the mean ± SD. *P* values for comparisons with control groups (NFD groups) are denoted as * *p* < 0.05 and ** *p* < 0.01, and those for comparisons with HFD groups are denoted as ^#^ *p* < 0.05, ^##^ *p* < 0.01, and ^###^ *p* < 0.001.

The protein levels of lipolysis-associated proteins, such as ATGL, p-HSL, and PLIN1, were significantly increased in both the pretreatment 5-*u*RCK- or ellagic acid-supplemented groups compared with those in the HFD groups (Figure 12).

### 2.17. Protein Expression Levels for Browning-Related Proteins in Subcutaneous ingWAT

**Figure 13:**
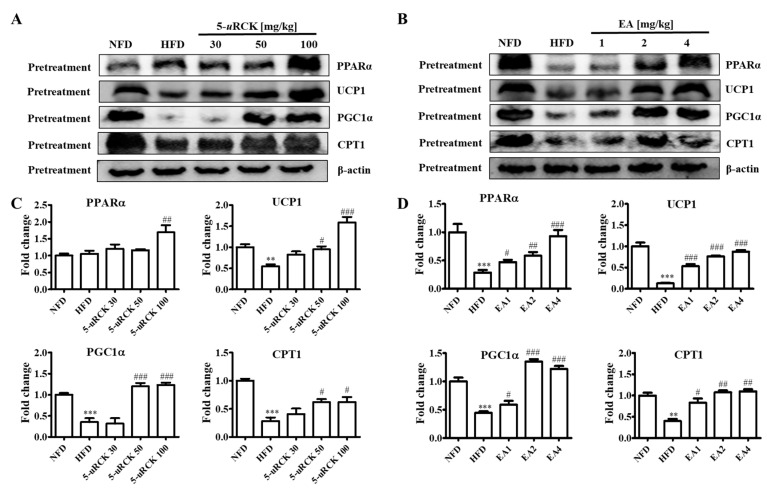
Effect of 5-*u*RCK and ellagic acid supplementation on fat browning-associated protein expression in HFD-fed mice inguinal white adipose tissue (ingWAT). Effects of 5-uRCK (**A**) and ellagic acid (**B**) on PPARα, UCP1, PGC1α, and CPT1 expression in the ingWAT and assessed by Western blot analysis. Each value (**C**,**D**) was normalized to β-actin and expressed as the mean ± SD. *P* values for comparisons with control groups (NFD groups) are denoted as ** *p* < 0.01 and *** *p* < 0.001, and those for comparisons with HFD groups are denoted as ^#^ *p* < 0.05, ^##^ *p* < 0.01, and ^###^ *p* < 0.001.

As shown in Figure 13, inguinal white adipose tissue expression of thermogenic proteins such as UCP1 and PGC1α was significantly reduced in the mice fed the HFD compared to those fed the NFD. UCP1 and PGC1α protein levels were significantly increased in the 5-*u*RCK- or ellagic acid-supplemented groups compared with those in the HFD groups. The expression of CPT1 significantly increased in both the 5-*u*RCK and ellagic acid groups compared with the HFD group. Additionally, the protein expression of PPARα was markedly increased in both the 5-*u*RCK and ellagic acid-pretreated groups.

## 3. Discussion

Obesity is characterized by increased adipose tissue mass that results from increases in both fat cell number and fat cell size. Adipose tissue is a dynamic organ that plays an important role in energy balance and changes in mass according to the metabolic requirements of the organism [26]. Excess energy intake and reduced energy expenditure result in abnormal excessive growth of WAT, which can lead to the development of obesity [29]. Furthermore, obesity is associated with an increased risk for the development of numerous metabolic complications. Many studies have indicated that phytochemicals have the potential to inhibit the differentiation of preadipocytes, reduce adipose tissue mass, stimulate lipolysis, and induce the apoptosis of existing adipocytes [30,31]. Our previous studies showed that the administration of 5-*u*RCK and ellagic acid to HFD mice significantly decreased body and fat weight gain in mice via adipogenic and lipogenic gene expression [21,22,23,24,25]. Therefore, the present study demonstrates the lipolytic and thermogenic activities of 5-*u*RCK and ellagic acid in adipocytes both in vitro and in vivo.

In the in vitro study results, GPDH activity was significantly decreased in mature 3T3-L1 adipocytes treated with 5-*u*RCK or ellagic acid compared with untreated control cells (Figure 2A,B). Since GPDH is an important enzyme for TG synthesis [32], it is suggested that 5-*u*RCK and ellagic acid suppress adipocyte differentiation. Thus, a reduction in GPDH activity may slow the de novo synthesis of fatty acids and TGs as well as inhibit lipogenesis in mature adipocytes [33]. Consequently, 5-*u*RCK and ellagic acid inhibited TG content after incubation for 3 days (Figure 2C,D). Furthermore, we showed that 5-*u*RCK (EC_50_ = 11.99 μg/mL) and ellagic acid (EC_50_ = 0.49 μM) treatment increased lipolysis and glycerol release in fully differentiated adipocytes by approximately 60% over control cells.

In the in vivo study results, the weight of eWAT in the 5-*u*RCK- and ellagic acid-treated groups was significantly decreased (post-treatment) (Figure 3, Figure 4 and Figure 5) and inhibited (pretreatment) (Figure 9 and Figure 10) compared to that in the HFD-fed groups. These results suggest that the 5-*u*RCK- and ellagic acid-mediated decrease in body weight was due to a reduction in adipose tissue weight independent of food intake. In previous studies, we elucidated the detailed mechanisms of adipogenesis by 5-*u*RCK and ellagic acid by regulating the expression of lipogenesis-related proteins in WAT [21,22,23,24]. In these studies, supplementation of the diet with 5-*u*RCK and ellagic acid ameliorated body weight and body fat gain of mice on an HFD independent of food intake. Adipogenesis is regulated by several transcriptional factors, such as SREBP, C/EBPs, and PPARγ. Furthermore, it is involved in the sequential mRNA expression of adipocyte-specific proteins such as adipocyte fatty acid–binding protein (aP2) and FAS. Our previous study demonstrated that 5-*u*RCK and its main active compound ellagic acid decreased lipid accumulation and expression levels of PPARγ, C/EBPα, SREBP-1c, ACC, and FAS to the greatest extent in an HFD-fed mouse model [22]. Moreover, 5-*u*RCK and ellagic acid treatment significantly increased the expression of AMPK, p-AMPK, p-HMGCR, and INSIG-1 and decreased the expression of HMGCR and mSREBP-2 in a HCD-induced hypercholesterolemia rat model [24]. Therefore, in the present study, the antiobesity effects of 5-*u*RCK and ellagic acid were evaluated by measuring lipolytic activity and thermogenesis in HFD-induced obese animal models. Furthermore, we tried to express the antiobesity effects of 5-*u*RCK and ellagic acid in an HFD-induced obese model and tried to establish if there is any superiority in preventive (pretreatment) or therapeutic (post-treatment) approach. We have realized that the pretreatment was superior to post-treatment in terms of lipolysis and thermogenesis with statistically higher ATGL and PLIN levels (marker of lipolysis), and UCP1 and PGC1α levels (marker of thermogenesis). However, both post-treated and pretreated mice showed significant reductions in body weight and adipose tissue mass compared to the HFD-fed mice. As a conclusion, 5-*u*RCK and ellagic acid were very effective in preventing and ameliorating HFD-induced obese and the both pretreatment and post-treatment use were superior in decreasing body weight.

In adipose tissue, AMPK directly phosphorylates ACC and ATGL, leading to the suppression of fatty acid synthesis as well as the promotion of fatty acid oxidation resulting from the diminished inhibition of CTP1 [34]. ATGL and HSL are major enzymes involved in the breakdown of TG in adipose tissue [35]. ATGL is the rate-limiting enzyme for lipolysis that hydrolyzes TG to diglycerides, whereas HSL is a multifunctional enzyme that has broad substrate specificity with a preference for diglycerides and needs to be phosphorylated for lipolysis [36]. It has been demonstrated that in the basal state, TG storage and fatty acid release are mainly influenced by the expression of ATGL [37]. In addition, perilipin, which is located on the surface of adipocyte lipid droplets, is a key protein in lipolysis regulation [38]. In our study, 5-*u*RCK and ellagic acid obviously increased ATGL and p-HSL expression levels in eWAT, which suggested that 5-*u*RCK and ellagic acid could indeed promote lipolysis in eWAT (Figure 7 and Figure 12).

In addition, the endogenously produced monounsaturated lipid oleoylethanolamide was demonstrated to increase epididymal adipose tissue lipolysis in a PPARα-dependent manner [39]. This indicates that, in addition to synthetic or natural compounds, naturally occurring lipids with ligand properties toward PPARs might be important modulators of lipase expression and lipolysis in adipose tissue. Similarly, we showed that 5-*u*RCK and ellagic acid treatment significantly elevated the expression of PPARα in inguinal white adipose tissue from HFD-fed mice (Figure 8 and Figure 13).

In adipose tissue, PPARα upregulates the expression of fatty acid oxidation enzymes, such as CPT1 and UCP1, which are associated with thermogenesis and energy balance [40,41]. BAT specializes in energy expenditure through an induction of UCP1 expression [42,43]. To date, the expression of UCP1 has been found to be regulated by the transcriptional factors of PR domain containing 16 (PRDM16) and PGC1α [44]. The activation of BAT contributes to whole-body energy expenditure and therefore is an attractive drug target for obesity treatment. However, the small amount of BAT in human adults certainly limits its usefulness in an antiobesity effect, utilizing BAT activity. Therefore, the browning of WAT is another attractive target for obesity treatment. Thus, reduction in visceral fat and the induction of brown-like adipocytes in WAT could prevent lifestyle-related diseases. Furthermore, lipolysis releases FFAs that are promptly eliminated by β-oxidation, and the rest enter the blood circulation [45]. However, an increase in lipolysis releases excessive FFAs that leads to lipotoxicity by impairing cellular signaling and function, resulting in decreased insulin sensitivity [46,47]. The 5-*u*RCK and ellagic acid treatment increased the protein levels of PPARα, CPT1, UCP1, and PGC1α in our in vivo experiment, suggesting that 5-*u*RCK and ellagic acid may exert antiobesity effects by increasing fatty acid β-oxidation and energy expenditure in adipocytes (Figure 8 and Figure 13). UCP1 expression levels were significantly increased in the 5-*u*RCK groups post-treated with low concentration (30–50 mg/kg), whereas the UCP1 levels did not increase significantly in the 5-*u*RCK post-treatment group treated with high concentration (100 mg/kg). Although UCP1 levels did not increase in the high concentration treated group, we could find that there was no significant difference when comparing UCP1 levels with the normal control group (NFD). This result is considered not to be the result of side effects caused by a high concentration treatment of 5-*u*RCK, and it can be hypothesized that there is a possibility that it may be a time point specific result according to the timing of tissue sampling. Therefore, much remains unclear regarding the development of beige adipocytes. However, it is certain that cold exposure and some other stimuli lead to the appearance of UCP1-positive adipocytes that share an *Myf5^−/−^* origin with white adipocytes in WAT depots of mice [48,49]. The diet-induced conversion of WAT to brown or beige adipocytes is a potentially valuable weight loss strategy because these cells dissipate metabolic energy as heat [50]. An ectopic expression of UCP1 in WAT is induced by β3-agonists and results in resistance to HFD-induced obesity [51]. Thus, compounds inducing UCP1 in WAT may be useful therapeutic agents for the treatment of obesity. Interestingly, the administration of ellagic acid to cold-exposed mice and HFD-fed mice induced the browning of WAT, as confirmed by the increase in UCP1 expression [52,53]. In the present study, we confirmed that an intake of 5-*u*RCK and its main active compound ellagic acid reduced the size of adipocytes and enhanced the production of UCP1 and PGC1α in subcutaneous ingWAT by immunoblot analyses. These results indicate that 5-*u*RCK and ellagic acid contain factors that induce browning of adipocytes, at least under our experimental conditions.

We also analyzed the effects of 5-*u*RCK and ellagic acid on the development of fatty liver, which is strongly associated with obesity [54]. Defects in fat metabolism are responsible for the pathogenesis of hepatic steatosis, which may be due to an imbalance in energy consumption and its combustion, resulting in lipid storage [55]. Additionally, the livers of HFD-induced obese rodents exhibited an accumulation of numerous fatty droplets, which is a typical sign of fatty liver, and the liver weights were significantly higher in the HFD-fed group than in the NFD-fed group [56,57]. Our results indicated that the continuous consumption of an HFD may play a role in the development of hepatic steatosis associated with obesity and that 5-*u*RCK and ellagic acid exhibit a hepatoprotective effect, as indicated by improvements in liver weight (Figure 4, Figure 5, Figure 9 and Figure 10) and hepatic fatty droplets (Figure 6 and Figure 11). Furthermore, serum AST and ALT levels are clinically and toxicologically important indicators [58] and increase as a result of liver damage caused by toxicants or disease conditions. In the HFD-fed group, the AST and ALT levels were significantly elevated relative to those in the NFD-fed group and were improved by 5-*u*RCK and ellagic acid supplementation (Table 1, Table 2, Table 3 and Table 4). These results suggest that 5-*u*RCK and ellagic acid supplementation may attenuate the development of hepatic steatosis and that ellagic acid is potentially effective in ameliorating fatty liver in HFD-induced obese mice.

Ellagic acid is present in raspberries, including *R. coreanus*, strawberries, walnuts, and pomegranate. As with other polyphenols, ellagic acid has chemoprotective activities with growth-inhibiting and apoptosis-promoting properties in cancer cells [59]. Ellagic acid also suppressed the secretion of resistin, an adipocytokine, by involving the degradation of intracellular resistin protein in adipocytes [60]. Ellagic acid is a phenolic compound found in fruits including grape juice (10.2 mg/100 g), grape wine (5.6 mg/100 g), blueberries (0.9 mg/100 g), blackberries (42.4 mg/100 g), raspberries (17.9 mg/100 g), and strawberries (919.8 mg/100 g) [61]. Our previous studies reported that the ellagic acid content of 5-*u*RCK was approximately 18–21 mg/g [24,62]. The typical dietary intake of ellagic acid in humans is approximately 12 mg/day if 600 mg 5-*u*RCK is eaten [24,25]. In the present study, we found that 1–4 mg/kg ellagic acid treatment profoundly reduced body weight and fat weight gain in HFD-fed mice. This dose (1–4 mg/kg in mice) of ellagic acid is equivalent to a human dosage of a dietary intake of approximately 400–800 mg/day of 5-*u*RCK subject to absorption [24,25]. In this study, 5-*u*RCK and ellagic acid were administered at 30–100 mg/kg and 1–4 mg/kg, respectively, which are the same doses used previously.

Adipose tissue serves not only as an organ for energy storage but also as an endocrine organ by releasing various inflammatory cytokines, such as tumor necrosis factor α (TNFα) and interleukin (IL)-6 [63,64,65]. Proinflammatory molecules produced by adipose tissue have been implicated as active participants in the development of inflammation and the increased risk of obesity-related insulin resistance [66,67,68]. An increased production of monocyte chemoattractant protein 1 (MCP1), interferon (IFN) α, IFNβ, TNFα, and IL-6 in adipose tissue has been reported in animal models of obesity [64,65,66,67]. Accordingly, therapeutic agents that attenuate proinflammatory cytokines may prove useful in the medical management of obesity-induced inflammation. Therefore, we are planning to study the association and effect of obesity and inflammatory cytokines with 5-*u*RCK and ellagic acid, which proved the antiobesity effect. The potential anti-inflammatory role of 5-*u*RCK and ellagic acid awaits further investigation. The cytotoxicity of extracts may be one factor involved in lipid metabolism. We have tested and reported the cytotoxicity and safety of 5-*u*RCK extracts in various cells and animal models through similar studies. As a result, we have not observed side effects in these extracts at concentrations up to 300 μg/mL in cells or 150 mg/kg in vivo [21,22,23,24]. Although this study did not provide direct toxicity results, it is expected that there will be no cytotoxicity according to the results of our previous report. Furthermore, in vivo and in vitro studies have limited application to humans. Therefore, future clinical trials will need to demonstrate the safety and efficacy of this drug for human obesity treatment in the future.

## 4. Materials and Methods

### 4.1. Reagents

3-Isobutyl-1-methylxanthine (IBMX), dexamethasone (DEX), insulin, 3-(4,5-dimethylthiazol-2-yl)-2,5-diphenyltetrazolium (MTT), Oil Red O, dimethyl sulfoxide (DMSO), and ellagic acid (EA) were purchased from Sigma Chemical Co. (St. Louis, MO, USA). Dulbecco’s modified Eagle’s medium (DMEM), bovine serum albumin (BSA), and fetal bovine serum (FBS) were purchased from Invitrogen, Inc. (Grand Island, NY, USA). All other chemicals were of analytical reagent grade.

### 4.2. Preparation of Extracts

The unripe *R. coreanus* (*u*RCK, specimen voucher number: BT-URCK001) fruits used in this study were collected (June 2018) in Gwangyang County (Jeollanam-do, Korea) and authenticated by Dr. Kim at B&Tech, Gwangju, South Korea. *u*RCK (2.5 kg) was extracted using 20 volumes of 5% ethanol at 100 for 4 h, as described in our previous study [21,22,23,24,25,62,69]. The extracted solution was then filtered, concentrated with an evaporator under vacuum, and freeze-dried. The dry matter content of the lyophilized samples was determined by drying at 105 °C to a constant mass. Normally, 20.4 g of dried powder could be obtained from 100 g of unripe *R. coreanus*.

### 4.3. Isolation of Ellagic Acid

The dried 5-*u*RCK (30 g) was suspended in distilled water and successively divided with *n*-hexane (3 × 500 mL), chloroform (CHCl_3_, 3 × 500 mL), ethyl acetate (EtOAc, 3 × 500 mL), and *n*-butanol (BuOH, 3 × 500 mL). The ethyl acetate fraction of 5-*u*RCK (18 g) was loaded onto a silica gel column (length: 32 cm, diameter: 2 cm, Merck, Darmstadt, Germany) and eluted with a stepwise gradient of chloroform, ethyl acetate, and methanol. The collected active subfractions were further separated by preparative thin layer chromatography (TLC), since the active isolate was not pure. It was again purified on an LH-20 column (length: 20 cm, diameter: 1.5 cm) using methanol as the eluant. The isolated compound was pure by high-performance liquid chromatography (HPLC). The amounts of ellagic acid were analyzed with HPLC and compared with a standard preparation of ellagic acid produced with our previously reported standard method [21,22,23,24,25,62].

### 4.4. Cell Culture

The 3T3-L1 preadipocytes were purchased from ATCC (American Type Culture Collection, CL-173, Manassas, VA, USA) and cultured in DMEM/high glucose supplemented with 10% newborn calf serum and 1% penicillin and streptomycin, which was defined as complete media, in a CO_2_ incubator at 37 °C. Preadipocytes were cultured and differentiated into adipocytes. Briefly, preadipocytes were cultured in complete medium. Two or three days after reaching confluence, cells were stimulated with differentiation medium (MDI) prepared by adding 1 μM dexamethasone, 0.5 mM 3-isobutyl-1-methyxanthine (IBMX), and 10 μg/mL insulin to complete medium (day 0). After two days (day 2), the cells were stimulated with 10 μg/mL insulin in complete medium. After three days (day 5), the differentiation media were replaced with complete media. The cell culture media were changed every 2 days until full differentiation, that is, up to 9 days (day 14). To investigate the effect of 5-*u*RCK or ellagic acid on lipolysis in mature 3T3-L1 adipocytes, cells were maintained in the presence or absence of 5-*u*RCK or ellagic acid from day 14 to day 17 (72 h) in six-well plates.

### 4.5. Lipid Accumulation, GPDH Activity, and Triglyceride Measurement

For lipid accumulation, Oil Red O staining was performed for 3T3-L1 cell lines. Mature adipocytes were incubated in complete media, with or without 5-*u*RCK or ellagic acid, for 72 h, and the cells were fixed with 4% formaldehyde for 20 min. Then, cells were washed with 60% isopropanol and fully dried. Oil Red O working solution was added, and cells were maintained at 30 min at room temperature. Mature fat cells were stained with Oil Red O solution. Excess stain was removed by washing thoroughly with distilled water. Fully differentiated mature fat cells were photographed using an inverted microscope (Nikon Instrument, Melville, NJ, USA). Differentiation was monitored using microscopy and quantified via elution with 100% isopropanol. Optical density (OD) measurements were obtained at 490 nm. GPDH activity was measured with a GPDH activity assay kit (Takara Bio Inc., Shiga, Japan) in accordance with the manufacturer’s instructions. Total triglyceride content in the mature adipocytes was measured; 100% isopropanol was added to mature adipocytes and incubated at room temperature for 10 min followed by measurement of absorbance at 630 nm using a TG assay kit (Asan pharmaceuticals, Seoul, Korea). Values for TG levels were expressed as a percentage of the control.

### 4.6. Measurement of Free Glycerol Release

The matured adipocytes were gently washed once with phosphate-buffered saline (PBS) and then incubated with DMEM (no phenol red and FBS) containing or without 5-*u*RCK or ellagic acid for 24 h. The culture medium of samples was collected, and glycerol levels in the media were assayed using a glycerol kit (Cayman Chemical Company, Ann Arbor, MI, USA) according to the manufacturer’s instructions.

### 4.7. Animals

Specific pathogen-free (SPF) grade healthy male four-week-old C57BL/6 mice weighing 18–21 g each were purchased from Central Lab Animal, Inc. (Seoul, Republic of Korea). Animals were maintained at a constant room temperature of 22 ± 2 °C with a humidity level of 50 ± 5% and with free access to water and food under a 12:12 h light:dark cycle (lights on at 8:00 am). The animals were acclimatized for 4 days before the beginning of the experiments. All efforts were made to minimize animal suffering and to reduce the number of animals used. The experiment was conducted according to the International Guidelines for the Care and Use of Laboratory Animals [70] and was approved by the institutional animal care and use committee (IACUC) of the Bioresources and Technology (B&Tech, Gwangju, Korea) Co., Ltd., Republic of Korea (Approval number: BT-004-2018, 9 July 2018). When the experiment began, all mice were anesthetized with isoflurane and then sacrificed by cervical dislocation in accordance with the IACUC guidelines.

### 4.8. Experimental Groups and Administration

The mice were randomly assigned to each treatment group consisting of 8–9 mice each (Table 5). The normal-fat diets consisted of 10% kcal from fat (Research Diets, D12450B, New Brunswick, NJ, USA). The HFD consisted of 60% kcal from fat (Research Diets, D12492, New Brunswick, NJ, USA). 5-*u*RCK (30, 50, and 100 mg/kg) or ellagic acid (1, 2, and 4 mg/kg) of 0.2 mL, dissolved in physiological saline solution, was orally administered using a stainless oral sonde (Jungdo-BNP, Seoul, South Korea), and the control rats received the same volume of saline solution. This dosage was calculated from an amount of 600 mg of 5-*u*RCK extract per day per 60 kg of human body weight. Food and water intake were measured every day, and body weight was measured once every two days. The feed efficiency ratio (FER) was calculated as follows: FER = *G* × *F*^−1^, where *G* is the weight gain (g) and *F* is the consumption (g) of dry matter from the feed [21,22,23,24].

### 4.9. Preparation of Blood Samples and Biochemical Characterization

At the end of the experimental period, the mice were fasted for 12 h prior to sacrifice. Blood samples were collected from the orbital venous plexus of mouse eyes and centrifuged at 1000× *g* and 4 °C for 15 min. Plasma was stored at −70 °C prior to the experiments. The concentrations of serum TC, triglyceride (TG), high-density lipoprotein cholesterol (HDL-C), low-density lipoprotein cholesterol (LDL-C), and glucose were measured using an Alere cholesterol LDX^®^ system (Cholestech LDX, Hayward, CA, USA). Aspartate aminotransferase (AST) and alanine aminotransferase (ALT) assays were performed with a kit according to the manufacturer’s instructions (Asan Pharm, Seoul, Korea). CRE, amylase, UA, and LDH assays were carried out using commercial kits in an automated biochemical analyzer (DRI-CHEM NX500i, FUGI-FILM, Tokyo, Japan). After blood collection, the adipose tissue (epididymal fat, perineal fat) and liver were removed from the mice and immediately weighed.

### 4.10. Histological Analysis of the Liver and Adipose Tissue

The epididymal adipose tissue was fixed in 4% formaldehyde and stored at −80 °C. Six-micron-thick sections of frozen adipose tissues were prepared using a cryomicrotome and stained with H&E. The liver was collected and fixed in PBS containing 4% formalin and embedded in paraffin, and five-micron-thick sections were stained with H&E. The images were acquired by light microscopy (Olympus BX51, Tokyo, Japan), and we analyzed the images with MetaMorph^®^ microscopy automation and image analysis software (Molecular Devices, Sunnyvale, CA, USA). The average adipocyte size of the fat pad was quantified by capturing four independent fields per blinded slice using ImageJ software (National Institutes of Health, Bethesda, MD, USA) and expressed in μm^2^/adipocyte.

### 4.11. Protein Extraction and Immunoblot Assays

Samples of epididymal or inguinal white adipose tissues were washed three times with cold PBS before being lysed in radioimmunoprecipitation (RIPA) lysis buffer (10 mmol/L Tris-HCl, pH 7.5, 1% NP-40; 0.1% sodium deoxycholate, 0.2% SDS, 150 mmol/L NaCl, and 1 mmol/L EDTA) supplemented with 1× protease and phosphatase inhibitor cocktail (Thermo, Fremont, CA, USA) on ice. The separated proteins were transferred onto a nitrocellulose membrane. The anti-ATGL (1:100, #2138), anti-HSL (1:200, #4107), anti-p-HSL (1:200. Ser563, #4139), anti-perilipin-1 (1:100, #3467), anti-UCP1 (1:100, #14670), anti-CPT1 (1:100), PGC1α (1:100, #4259), and anti-β-actin (1:3000, #4967) antibodies were obtained from Cell Signaling Technology (Danvers, MA, USA). The monoclonal anti-PPARα antibody (1:200, ab191226) and secondary antibodies (1:10,000) were obtained from Abcam (Cambridge, MA, USA). Immunoreactive protein bands were visualized using a ChemiDoc XRS+ System (Bio-Rad) and quantified with Gel Pro Analyzer software (Silk Scientific, Inc., Orem, UT, USA). The internal control, β-actin, was used to normalize differences due to loading variations.

### 4.12. Statistical Analysis

Data are presented as the mean ± standard deviation (SD) or standard error (SE) from three independent experiments with replication. Data were statistically evaluated using Student’s *t*-test or one-way analysis of variance (ANOVA) with GraphPad Prism 5 version 5.01 for Windows (GraphPad, Inc., San Diego, CA, USA) software programs. Statistical significance was indicated when *p* < 0.05.

## 5. Conclusions

Our main findings indicate that 5-*u*RCK and ellagic acid increase adipose tissue lipolysis as well as the expression of the major triacylglycerol lipases ATGL and p-HSL. Here, we present strong evidence suggesting that the positive effects of 5-*u*RCK and ellagic acid on lipolysis and lipase expression require the proper functioning of the lipid sensor PPARα. Furthermore, PPARα upregulates the expression of fatty acid oxidation enzymes, such as CPT-1, UCP1, and PGC1α, which are associated with the browning of WAT and energy balance. Our data indicate that 5-*u*RCK and ellagic acid are important positive modulators of adipocyte browning and the content of the major proteins UCP1 and PGC1α through a PPARα-dependent mechanism. In conclusion, lipid metabolism was improved via the induction of adipocyte lipolysis, fatty acid oxidation, and the expression levels of key thermogenesis proteins by 5-*u*RCK and ellagic acid in vitro and in vivo.

## Figures and Tables

**Table 1:** Effect of 5-*u*RCK on blood biochemical parameters in HFD-fed mice.

Biomarker	NFD	HFD	5-*u*RCK 30	5-*u*RCK 50	5-*u*RCK 100
TG (mg/dL)	117.50 ± 6.72	160.50 ± 5.55 ***	161.00 ± 3.27	152.10 ± 7.85	143.90 ± 3.94 ^#^
TC (mg/dL)	115.80 ± 3.52	210.30 ± 7.22 ***	205.30 ± 4.10	197.50 ± 3.92	187.70 ± 4.40 ^#^
HDL-C (mg/dL)	48.59 ± 1.52	23.25 ± 1.14 ***	42.62 ± 1.77 ^###^	47.33 ± 0.72 ^###^	44.86 ± 4.29 ^###^
LDL-C (mg/dL)	67.51 ± 4.17	139.00 ± 6.26 ***	121.00 ± 6.01	119.90 ± 1.95 ^#^	109.20 ± 5.02 ^##^
AST (U/I)	127.10 ± 6.18	235.50 ± 24.62 ***	143.60 ± 8.44 ^##^	119.90 ± 3.88 ^###^	123.90 ± 10.26 ^##^
ALT (U/I)	23.00 ± 1.09	58.50 ± 2.85 ***	53.63 ± 5.93	44.90 ± 7.33	41.90 ± 2.73 ^###^
CRE (mg/dL)	0.21 ± 0.03	0.56 ± 0.04 ***	0.25 ± 0.01 ^###^	0.28 ± 0.01 ^###^	0.28 ± 0.02 ^###^
Amylase (U/I)	839.90 ± 25.31	1241.00 ± 47.45 ***	1045.00 ± 16.18 ^##^	1017.00 ± 45.18 ^##^	906.10 ± 19.38 ^###^
UA (U/I)	1.55 ± 0.12	3.30 ± 0.46 **	3.06 ± 0.11	1.46 ± 0.04 ^###^	1.44 ± 0.04 ^###^
LDH (U/I)	213.60 ± 13.49	334.60 ± 23.44 **	235.90 ± 21.26 ^##^	223.30 ± 13.91 ^###^	196.40 ± 25.00 ^###^
Glucose (mg/dL)	189.30 ± 13.08	268.20 ± 33.67 *	255.50 ± 8.30	235.20 ± 10.57	236.10 ± 19.21

* *p* < 0.05, ** *p* < 0.01 and *** *p* < 0.001 vs. NFD group; ^#^ *p* < 0.05, ^##^ *p* < 0.01 and ^###^ *p* < 0.001 vs. HFD group. TG, triglyceride; TC, total cholesterol; HDL-C, high-density lipoprotein cholesterol; LDL-C, low-density lipoprotein cholesterol; AST, aspartate aminotransferase; ALT, alanine aminotransferase; CRE, creatinine; UA, uric acid; LDH, lactate dehydrogenase.

**Table 2:** Effect of ellagic acid on blood biochemical parameters in HFD-fed mice.

Biomarker	NFD	HFD	EA 1	EA 2	EA 4
TG (mg/dL)	101.31 ± 6.19	233.71 ± 22.01 ***	112.11 ± 7.30 ^###^	96.91 ± 4.26 ^###^	112.01 ± 7.57 ^###^
TC (mg/dL)	112.72 ± 5.45	175.01 ± 5.08 ***	161.61 ± 11.91	126.41 ± 6.42 ^###^	126.11 ± 3.28 ^###^
HDL-C (mg/dL)	46.22 ± 1.39	24.00 ± 2.39 ***	35.00 ± 1.53 ^##^	39.92 ± 3.45 ^##^	46.18 ± 3.78 ^###^
LDL-C (mg/dL)	69.31 ± 4.83	99.28 ± 5.50 **	88.43 ± 11.88	67.53 ± 6.99 ^##^	65.17 ± 3.91 ^###^
AST (U/I)	150.60 ± 15.79	272.41 ± 20.51 ***	154.01 ± 13.47 ^###^	144.40 ± 14.81 ^###^	149.31 ± 7.31 ^###^
ALT (U/I)	27.57 ± 2.28	90.45 ± 10.37 ***	56.29 ± 5.19 ^#^	39.71 ± 4.32 ^##^	34.71 ± 1.36 ^###^
CRE (mg/dL)	0.11 ± 0.01	0.38 ± 0.04 ***	0.22 ± 0.05 ^#^	0.19 ± 0.01 ^##^	0.19 ± 0.02 ^##^
Amylase (U/I)	603.01 ± 29.08	1059.01 ± 47.63 ***	690.70 ± 32.48 ^###^	680.31 ± 29.71 ^###^	615.61 ± 39.97 ^###^
UA (U/I)	1.59 ± 0.19	3.26 ± 0.27 ***	3.16 ± 0.12	3.16 ± 0.22	3.30 ± 0.05
LDH (U/I)	162.71 ± 23.92	402.91 ± 60.70 **	335.71 ± 25.31	321.61 ± 22.52	357.01 ± 26.22
Glucose (mg/dL)	225.62 ± 5.50	333.01 ± 25.20 **	210.71 ± 3.89 ^###^	190.91 ± 7.68 ^###^	118.71 ± 9.02 ^###^

** *p* < 0.01 and *** *p* < 0.001 vs. NFD group; ^#^ *p* < 0.05, ^##^ *p* < 0.01 and ^###^ *p* < 0.001 vs. HFD group. TG, triglyceride; TC, total cholesterol; HDL-C, high-density lipoprotein cholesterol; LDL-C, low-density lipoprotein cholesterol; AST, aspartate aminotransferase; ALT, alanine aminotransferase; CRE, creatinine; UA, uric acid; LDH, lactate dehydrogenase.

**Table 3:** Effect of 5-*u*RCK on blood biochemical parameters in HFD-fed mice.

Biomarker	NFD	HFD	5-*u*RCK 30	5-*u*RCK 50	5-*u*RCK 100
TG (mg/dL)	138.90 ± 7.57	215.50 ± 14.64 ***	154.90 ± 4.32 ^##^	147.90 ± 4.42 ^##^	148.60 ± 11.08 ^##^
TC (mg/dL)	103.00 ± 9.01	193.30 ± 3.98 ***	194. 30 ± 4.71	154.90 ± 13.15 ^#^	153.80 ± 3.06 ^###^
HDL-C (mg/dL)	45.17 ± 1.09	23.64 ± 2.05 ***	37.44 ± 3.53 ^##^	38.75 ± 0.59 ^###^	46.86 ± 2.09 ^###^
LDL-C (mg/dL)	80.89 ± 6.26	145.60 ± 3.80 ***	145.80 ± 2.35	121.20 ± 8.92 ^#^	111.90 ± 2.26 ^###^
AST (U/I)	118.00 ± 3.33	316.00 ± 7.97 ***	141.20 ± 11.14 ^###^	121.30 ± 3.94 ^###^	114.8 ± 12.92 ^###^
ALT (U/I)	26.38 ± 0.91	79.25 ± 3.14 ***	35.63 ± 1.00 ^###^	26.13 ± 2.11 ^###^	24.38 ± 1.31 ^###^
CRE (mg/dL)	0.20 ± 0.03	0.44 ± 0.04 ***	0.31 ± 0.01 ^##^	0.26 ± 0.02 ^###^	0.26 ± 0.02 ^###^
Amylase (U/I)	708.00 ± 32.70	982.10 ± 35.35 ***	1012.01 ± 22.67	914.50 ± 25.03	845.60 ± 31.84 ^#^
UA (U/I)	2.32 ± 0.10	4.62 ± 0.17 ***	2.21 ± 0.24 ^###^	1.81 ± 0.19 ^###^	2.11 ± 0.23 ^###^
LDH (U/I)	165.40 ± 12.20	429.00 ± 25.45 ***	249.60 ± 22.34 ^###^	217.90 ± 17.70 ^###^	178.70 ± 10.73 ^###^
Glucose (mg/dL)	173.80 ± 5.15	220.90 ± 7.93 ***	202.60 ± 5.82	208.80 ± 7.44	203.80 ± 9.14

*** *p* < 0.001 vs. NFD group; ^#^ *p* < 0.05, ^##^ *p* < 0.01 and ^###^ *p* < 0.001 vs. HFD group. TG, triglyceride; TC, total cholesterol; HDL-C, high-density lipoprotein cholesterol; LDL-C, low-density lipoprotein cholesterol; AST, aspartate aminotransferase; ALT, alanine aminotransferase; CRE, creatinine; UA, uric acid; LDH, lactate dehydrogenase.

**Table 4:** Effect of ellagic acid on blood biochemical parameters in HFD-fed mice.

Biomarker	NFD	HFD	EA 1	EA 2	EA 4
TG (mg/dL)	133.40 ± 11.53	190.01 ± 10.00 **	174.10 ± 4.38	138.40 ± 6.20 ^###^	129.61 ± 4.08 ^###^
TC (mg/dL)	122.30 ± 10.61	216.31 ± 4.21 ***	202.71 ± 3.18 ^#^	180.31 ± 4.63 ^###^	175.30 ± 3.44 ^###^
HDL-C (mg/dL)	50.10 ± 3.30	31.86 ± 1.90 ***	48.17 ± 1.47 ^###^	53.43 ± 1.20 ^###^	54.77 ± 1.84 ^###^
LDL-C (mg/dL)	60.46 ± 12.14	137.01 ± 3.96 ***	108.01 ± 3.51 ^###^	96.03 ± 5.07 ^###^	89.51 ± 4.74 ^###^
AST (U/I)	156.31 ± 3.44	249.01 ± 6.91 ***	191.40 ± 9.07 ^###^	189.41 ± 13.21 ^###^	184.30 ± 16.80 ^###^
ALT (U/I)	25.14 ± 2.15	58.86 ± 4.43 ***	49.71 ± 2.12	35.57 ± 2.10 ^###^	24.86 ± 2.09 ^###^
CRE (mg/dL)	0.21 ± 0.03	0.47 ± 0.02 ***	0.43 ± 0.03	0.35 ± 0.02 ^##^	0.25 ± 0.03 ^###^
Amylase (U/I)	716.70 ± 30.43	1198.01 ± 45.59 ***	921.30 ± 22.32 ^###^	911.41 ± 22.72 ^###^	899.00 ± 28.02 ^###^
UA (U/I)	2.40 ± 0.19	5.89 ± 0.40 ***	5.41 ± 0.50	4.41 ± 0.36 ^#^	2.87 ± 0.20 ^###^
LDH (U/I)	112.70 ± 15.12	474.70 ± 34.67 ***	407.20 ± 20.99	369.50 ± 19.53 ^#^	261.90 ± 16.81 ^###^
Glucose (mg/dL)	169.10 ± 6.99	255.40 ± 16.56 ***	203.30 ± 6.92 ^#^	198.60 ± 18.29 ^#^	179.40 ± 5.13 ^###^

** *p* < 0.01 and *** *p* < 0.001 vs. NFD group; ^#^ *p* < 0.05, ^##^ *p* < 0.01 and ^###^ *p* < 0.001 vs. HFD group. TG, triglyceride; TC, total cholesterol; HDL-C, high-density lipoprotein cholesterol; LDL-C, low-density lipoprotein cholesterol; AST, aspartate aminotransferase; ALT, alanine aminotransferase; CRE, creatinine; UA, uric acid; LDH, lactate dehydrogenase.

**Table 5:** Experimental group design.

	Test 1 (Post-Treatment)	Test 2 (Post-Treatment)	Test 3 (Post-Treatment)	Test 4 (Pretreatment)	Test 5 (Pretreatment)
Experimental Groups	Normal fat diet group (NFD) n = 9	Normal fat diet group (NFD) n = 8	Normal fat diet group (NFD) n = 8	Normal fat diet group (NFD) n = 8	Normal fat diet group (NFD) n = 8
	High-fat diet group (HFD)/16-weeks n = 9	High-fat diet group (HFD)/8-weeks n = 8	High-fat diet group (HFD)/8-weeks n = 8	High-fat diet group (HFD)/6-weeks n = 8	High-fat diet group (HFD)/6-weeks n = 8
	NFD + 5-*u*RCK 100 mg/kg/day group, n = 9	HFD + 5-*u*RCK 30 mg/kg/day group, n = 8	HFD + EA 1 mg/kg/day group, n = 8	HFD + 5-*u*RCK 30 mg/kg/day group, n = 8	HFD + EA 1 mg/kg/day group, n = 8
	HFD + 5-*u*RCK 30 mg/kg/day group, n = 9	HFD + 5-*u*RCK 50 mg/kg/day group, n = 8	HFD + EA 2 mg/kg/day group, n = 8	HFD + 5-*u*RCK 50 mg/kg/day group, n = 8	HFD + EA 2 mg/kg/day group, n = 8
	HFD + 5-*u*RCK 50 mg/kg/day group, n = 9	HFD + 5-*u*RCK 100 mg/kg/day group, n = 8	HFD + EA 4 mg/kg/day group, n = 8	HFD + 5-*u*RCK 100 mg/kg/day group, n = 8	HFD + EA 4 mg/kg/day group, n = 8
	HFD + 5-*u*RCK 100 mg/kg/day group, n = 9				

## Supplementary Materials

The following are available online, Figure S1: Effects of 5-*u*RCK on Liver coefficient (%) and epididymal adipose tissue coefficient (%) in HFD-induced obese mice, Figure S2: Ellagic acid ameliorates obesity in HFD-fed mice, Figure S3: Effects of 5-*u*RCK on Liver coefficient (%) and epididymal adipose tissue coefficient (%) in HFD-induced obese mice, Figure S4: Ellagic acid ameliorates obesity in HFD-fed mice.
