# Supplementary material for: Unripe Rubus coreanus Miquel Extract Containing Ellagic Acid Promotes Lipolysis and Thermogenesis In Vitro and In Vivo
Source: Molecules. 2020 Dec 16;25(24):5954. doi: 10.3390/molecules25245954 (PMC7766442; doi:10.3390/molecules25245954)
Supplement: Supplementary file 1 [file molecules-25-05954-s001.pdf]

molecules

Article

Unripe *Rubus coreanus* Miquel Extract Containing Ellagic Acid Promotes Lipolysis and Thermogenesis In Vitro and In Vivo

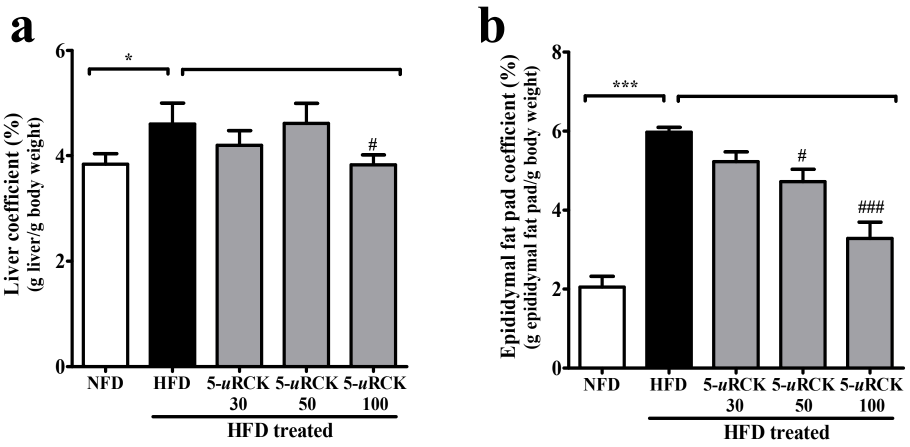

Figure S1. Effects of 5-*u*RCK on Liver coefficient (%) and epididymal adipose tissue coefficient (%) in HFD-induced obese mice. (**a**) Liver coefficient (%), which was calculated using the following formula: Liver tissue weight/body weight × 100 (%). (**b**) Epididymal fat pad coefficient (%), which was calculated using the following formula: Epididymal fat pad weight/body weight × 100 (%). The results are presented as the mean ± SD (n=8). *p<0.05, **p<0.01 and ***p<0.001 vs. NFD group; $^{\#}p$<0.05 and $^{\#\#\#}p$<0.001 vs HFD group.

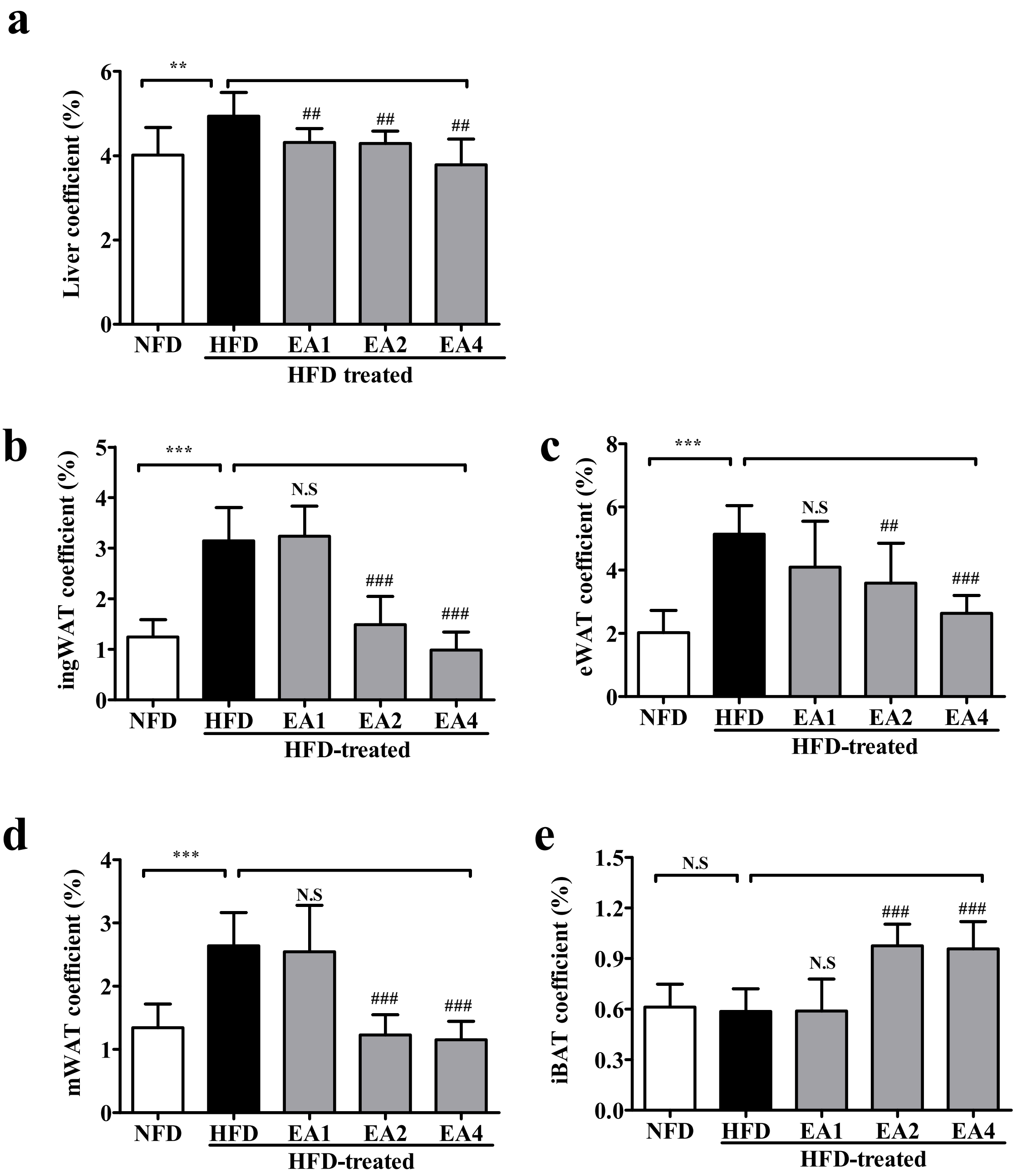

Figure S2. Ellagic acid ameliorates obesity in HFD-fed mice. (**a**) liver coefficients (%), (**b**) inguinal white adipose tissue coefficients (%), (**c**) epididymal white adipose tissue coefficients (%), (**d**) mesenteric white adipose tissue coefficients (%) and (**e**) interscapular brown adipose tissue coefficients (%). Tissue coefficients (%) represented as each tissue weight/body weight × 100 (%). The results are presented as the mean ± SD (n=8). $^{*}p<0.05$, $^{**}p<0.01$ and $^{***}p<0.001$ vs. NFD group; $^{\#}p<0.05$, $^{\#\#}p<0.01$ and $^{\#\#\#}p<0.001$ vs HFD group. N.S., not significant.

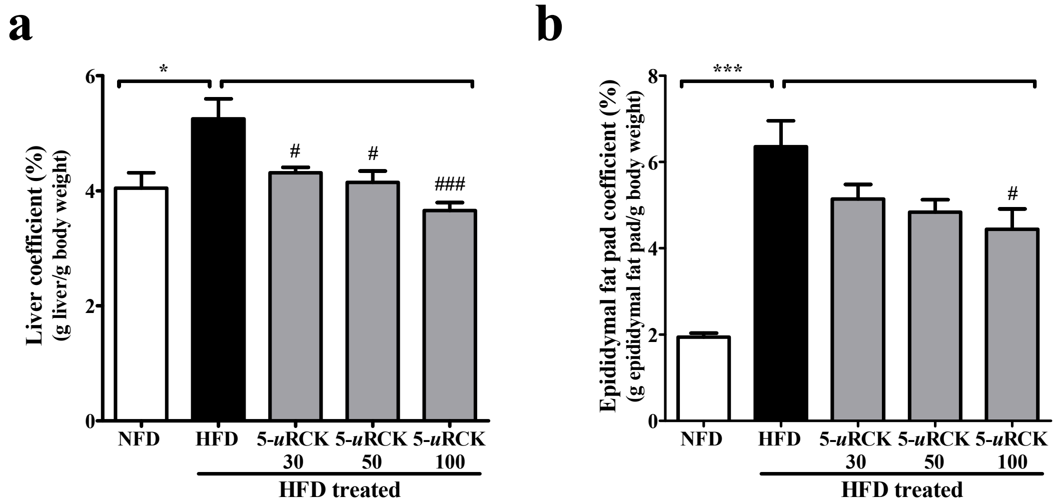

Figure S3. Effects of 5-*u*RCK on Liver coefficient (%) and epididymal adipose tissue coefficient (%) in HFD-induced obese mice (**a**) Liver coefficient (%), which was calculated using the following formula: Liver tissue weight/body weight × 100 (%). (**b**) Epididymal fat pad coefficient (%), which was calculated using the following formula: Epididymal fat pad weight/body weight × 100 (%). The results are presented as the mean ± SD (n=8). *p<0.05, **p<0.01 and ***p<0.001 vs. NFD group; #p<0.05 and ###p<0.001 vs HFD group.

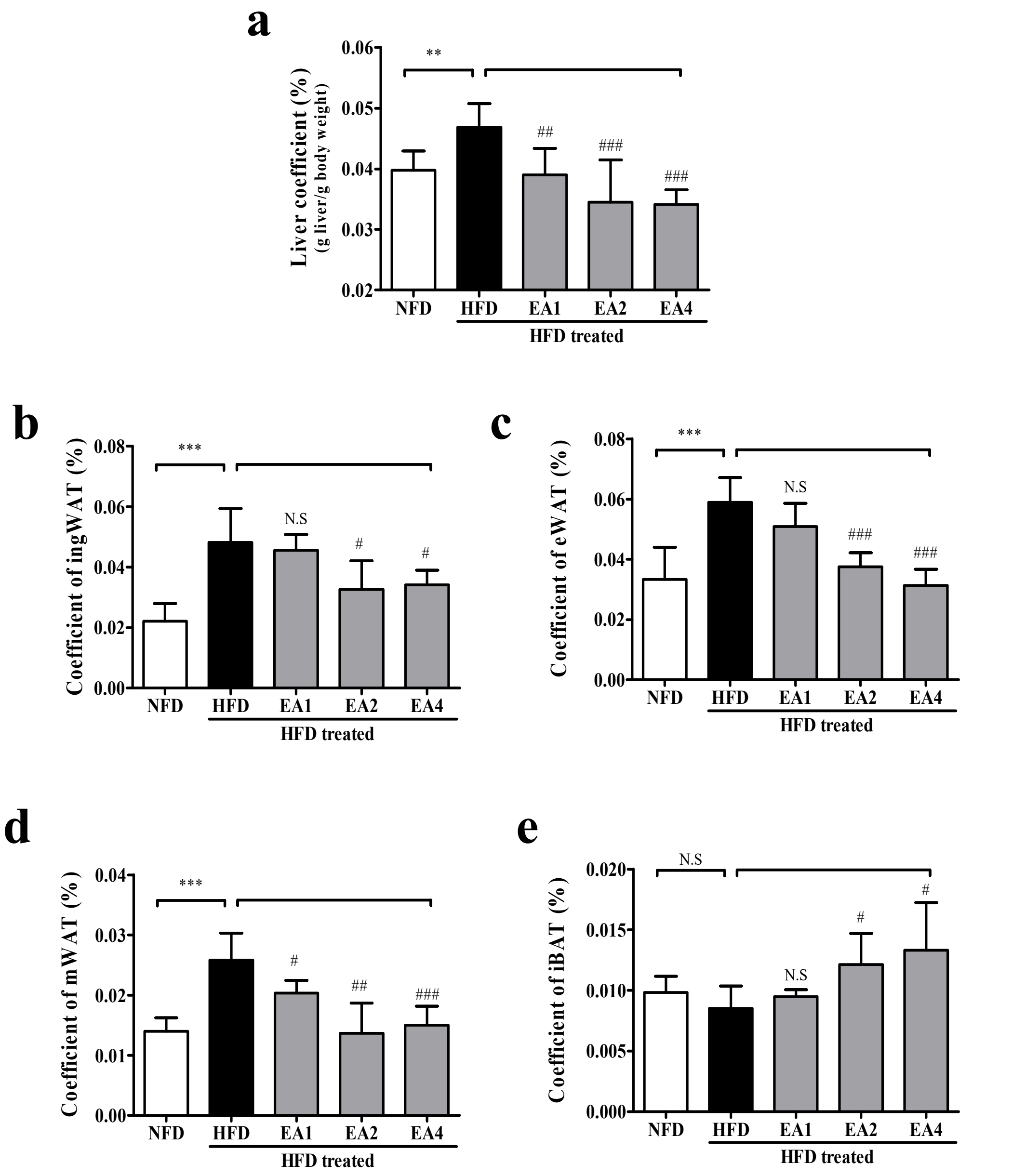

Figure S4. Ellagic acid ameliorates obesity in HFD-fed mice. (**a**) liver coefficients (%), (**b**) inguinal white adipose tissue coefficients (%), (**c**) epididymal white adipose tissue coefficients (%), (**d**) mesenteric white adipose tissue coefficients (%) and (**e**) interscapular brown adipose tissue coefficients (%). Tissue coefficients (%) represented as each tissue weight/body weight × 100 (%). The results are presented as the mean ± SD (n=8). $^{*}p<0.05$, $^{**}p<0.01$ and $^{***}p<0.001$ vs. NFD group; $^{\#}p<0.05$, $^{\#\#}p<0.01$ and $^{\#\#\#}p<0.001$ vs HFD group. N.S., not significant.
